# Mutation of the conserved late element in geminivirus *CP* promoters abolishes *Arabidopsis* TCP24 transcription factor binding and decreases H3K27me3 levels on viral chromatin

**DOI:** 10.1371/journal.ppat.1012399

**Published:** 2024-07-18

**Authors:** Jacqueline Williams, Elizabeth Regedanz, Natalia Lucinda, Alba Ruth Nava Fereira, Gabriela Lacatus, Mary Berger, Nels O’Connell, Tami Coursey, Jianhua Ruan, David M. Bisaro, Garry Sunter

**Affiliations:** 1 Department of Biology, The University of Texas at San Antonio, San Antonio, Texas, United States of America; 2 Department of Molecular Genetics, Center for Applied Plant Sciences, Center for RNA Biology, and Infectious Diseases Institute, The Ohio State University, Columbus, Ohio, United States of America; 3 Department of Biological Sciences, Northern Illinois University, DeKalb, Illinois, United States of America; 4 Department of Computer Science, The University of Texas at San Antonio, San Antonio, Texas, United States of America; Agriculture and Agri-Food Canada, CANADA

## Abstract

In geminiviruses belonging to the genus *Begomovirus*, coat protein (*CP*) expression depends on viral AL2 protein, which derepresses and activates the *CP* promoter through sequence elements that lie within the viral intergenic region (IR). However, AL2 does not exhibit sequence-specific DNA binding activity but is instead directed to responsive promoters through interactions with host factors, most likely transcriptional activators and/or repressors. In this study, we describe a repressive plant-specific transcription factor, *Arabidopsis thaliana* TCP24 (AtTCP24), that interacts with AL2 and recognizes a class II TCP binding site in the *CP* promoter (GTGGTCCC). This motif corresponds to the previously identified conserved late element (CLE). We also report that histone 3 lysine 27 trimethylation (H3K27me3), an epigenetic mark associated with facultative repression, is enriched over the viral IR. H3K27me3 is deposited by Polycomb Repressive Complex 2 (PRC2), a critical regulator of gene expression and development in plants and animals. Remarkably, mutation of the TCP24 binding site (the CLE) in tomato golden mosaic virus (TGMV) and cabbage leaf curl virus (CaLCuV) *CP* promoters greatly diminishes H3K27me3 levels on viral chromatin and causes a dramatic delay and attenuation of disease symptoms in infected *Arabidopsis* and *Nicotiana benthamiana* plants. Symptom remission is accompanied by decreased viral DNA levels in systemically infected tissue. Nevertheless, in transient replication assays CLE mutation delays but does not limit the accumulation of viral double-stranded DNA, although single-stranded DNA and CP mRNA levels are decreased. These findings suggest that TCP24 binding to the CLE leads to *CP* promoter repression and H3K27me3 deposition, while TCP24-AL2 interaction may recruit AL2 to derepress and activate the promoter. Thus, a repressive host transcription factor may be repurposed to target a viral factor essential for promoter activity. The presence of the CLE in many begomoviruses suggests a common scheme for late promoter regulation.

## Author summary

Geminiviruses are important pathogens of crop species world-wide. Yet, while it is understood that they co-opt host transcription and epigenetic machinery, relatively little is known about the regulation of viral gene expression programs. Here, we identify TCP24 as a repressive host transcription factor that binds the CLE within TGMV and CaLCuV *CP* promoters and demonstrate that it also interacts with viral AL2 protein, which is required to derepress and activate *CP* expression. In addition, we show that H3K27me3, a repressive epigenetic mark, is enriched on viral chromatin over the intergenic region encompassing the *CP* promoter. H3K27me3 is deposited by PRC2, a critical regulator of cell differentiation that is conserved among higher eukaryotes. However, how PRC2 is targeted and how targets are converted from transcriptionally inactive to active is incompletely understood. Providing a potential link between TCP24 and PRC2, we show that mutation of the TCP24 binding site (the CLE) in the *CP* promoters of TGMV and CaLCuV greatly diminishes H3K27me3 deposition on viral chromatin, reduces *CP* expression, and dramatically attenuates infection. Consistent with these results, we propose that TCP24 binding to the CLE leads to *CP* promoter repression and H3K27me3 deposition, while TCP24-AL2 interaction recruits AL2 to derepress and drive *CP* expression.

## Introduction

Geminiviruses are important plant pathogens that encapsidate circular single-stranded DNA (ssDNA) genomes in twin icosahedral particles [1,2]. Viral genomes are comprised of one or two ~2.5–3.0 kb ssDNAs that are replicated and transcribed by host polymerases and associated machinery. Upon entering the nucleus, genomic ssDNA is converted to a double-stranded DNA (dsDNA) replicative form (RF) that associates with histone octamers to form non-integrating minichromosomes. While these serve as replication and transcription templates, they can also be targeted by antiviral defenses that result in transcriptional gene silencing. As a result, both active and silenced viral chromosomes are present in infected hosts. Silenced viral chromatin exists in a highly condensed state with high levels of cytosine and histone 3 lysine 9 dimethylation (H3K9me2), characteristic of constitutive heterochromatin. In contrast, active viral chromosomes are relatively de-condensed, largely lack DNA methylation, and display histone marks typical of transcriptionally permissive euchromatin. These include H3K9 acetylation, H3K4me3, and H3K36me3. H3K27me3, a feature of facultative heterochromatin, is also present [3–7]. Thus, while geminiviruses are vulnerable to host epigenetic defenses, selected components of the epigenetic machinery are hijacked to regulate viral gene expression.

**Fig 1:**
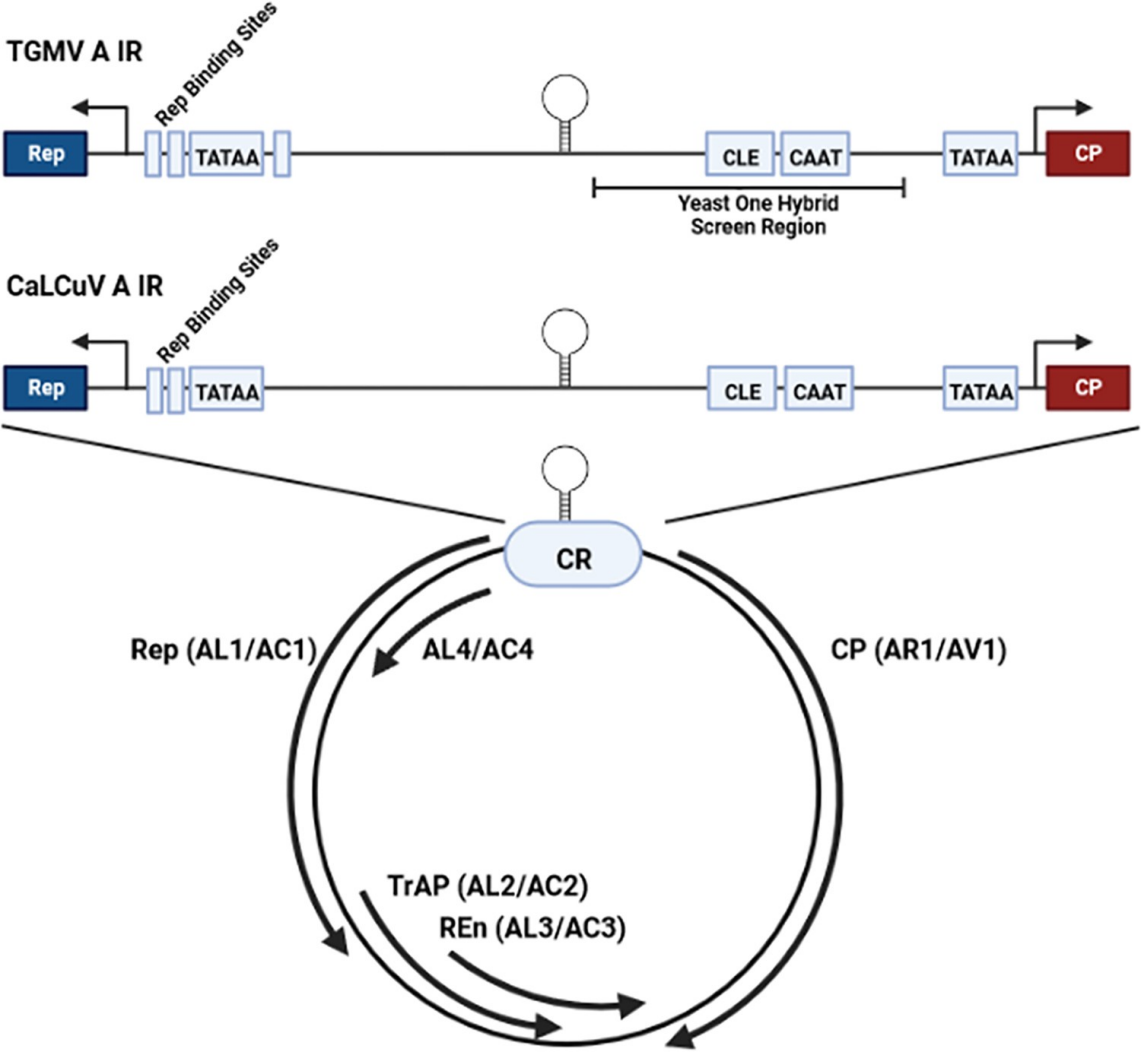
Organization of geminivirus DNA-A. The circular diagram depicts the dsDNA RF of a typical bipartite begomovirus (e.g., TGMV, CaLCuV), with expanded views of the intergenic region (IR). Viral genes, indicated by solid arrows, encode replication initiator protein (Rep/AL1/AC1); transcriptional activator protein (TrAP/AL2/AC2); replication enhancer; (REn/AL3/AC3); and coat protein (CP/AR1/AV1). AL4/AC4 is a pathogenicity factor encoded within the Rep ORF in a different reading frame. Within the IR, the common region (CR) present in both DNA-A and DNA-B is represented by a light blue ellipse. The replication origin core includes the conserved hairpin and Rep binding sites. The *Rep* and *CP* transcription start sites (TSS) are indicated by right angle arrows. The conserved late element (CLE), CAAT, and TATAA box sequences are indicated in light blue boxes. The region used in the yeast one-hybrid screen is also shown. Diagrams are not to scale.

Geminiviruses employ a strategy wherein early gene products enable expression of genes required later in the replication cycle. In bipartite members of the genus *Begomovirus* that have two genome components (DNA-A and DNA-B), such as tomato golden mosaic virus (TGMV) and cabbage leaf curl virus (CaLCuV), early genes encode the replication initiator protein (Rep/AL1/AC1) and replication enhancer (REn/AL3/AC3), both encoded in DNA-A. Late genes specify the coat protein (CP/AR1/AV1, encoded in DNA-A) and nuclear shuttle protein (NSP/BR1/BV1, encoded in DNA-B). The transcriptional activator protein and pathogenicity factor AL2 (AC2/TrAP), a delayed early gene product encoded in DNA-A, is required for expression of *CP* and *NSP* [8–10]. These genes are similarly regulated because their promoters lie within a portion of the ~300-bp viral intergenic region (IR) referred to as the common region (CR), a sequence that is nearly identical in DNA-A and DNA-B. In TGMV, AL2 has been shown to derepress and activate the *CP* promoter in mesophyll cells and protoplasts via sequence elements located between -125 to -60 bp upstream of the *CP* transcription start site (TSS) (Fig 1) [9]. AL2 also derepresses the *CP* promoter in phloem through a distal repressor element located outside the IR, between 1.5 to 1.2 kb upstream of the TSS [8,9]. More recent studies have shown that the CaLCuV *CP* promoter is similarly organized and regulated by AL2 [10].

AL2 has a strong acidic activation domain but does not exhibit sequence-specific DNA binding, including sequences involved in AL2-mediated activation and derepression [9,11]. Thus, it is believed to be directed by interaction with host factors, most likely transcriptional activators and/or repressors, that recognize specific sequences within AL2 responsive promoters. A similar mechanism is shared by several viral activators, including herpes simplex virus VP16, adenovirus E1A, and Epstein Barr virus EBNA-2 [12–15]. Indirect promoter targeting has the advantage of providing opportunities (via AL2 for geminiviruses) to alter host gene expression [16], which could reprogram the host to support virus infection and/or suppress host defenses. That TGMV and CaLCuV AL2 can derepress and activate a *CP* promoter integrated into host chromosomes lends support for this hypothesis [8,10]. Furthermore, AL2 proteins are functionally interchangeable with respect to *CP* promoter activation, at least among bipartite begomoviruses [17].

Attempts to identify conserved motifs diagnostic for host transcription factors within AL2-responsive promoters have met with limited success, although a sequence termed the conserved late element (CLE, GTGGTCCC) found in many begomovirus late promoters (i.e., *CP* and *NSP*), including those of TGMV and CaLCuV, was shown to be required for expression of the pepper hausteco yellow vein virus (PHYVV) *CP* promoter and for AL2-responsiveness of the TGMV *CP* promoter [18,19]. However, not all AL2-responsive begomovirus late promoters contain this element [20,21], and CLE deletion had no significant effect on AL2-mediated activation of the TGMV *CP* promoter in transient assays [9]. Because of this, and the absence of a known binding factor, the role of the CLE has remained an enigma.

That sequence elements within and upstream of the IR are involved in TGMV and CaLCuV *CP* promoter regulation suggests that AL2 interacts with several distinct host factors. However, to our knowledge, only one host protein that interacts with AL2 and geminivirus DNA has been identified. We previously found that a plant-specific transcription factor, *Arabidopsis thaliana* PEAPOD2 (AtPPD2, also known as TIFY4B) forms a complex with AL2 and sequences within the TGMV and CaLCuV IRs [22].

To extend our studies on the molecular mechanisms of begomovirus late gene expression, we searched for additional host factors. Here, we describe *Arabidopsis thaliana* TCP24 (AtTCP24), a repressive transcription factor that interacts with AL2 and specifically binds TGMV and CaLCuV *CP* promoter sequences via a class II TCP binding site that is identical to the CLE. TCP proteins comprise a family of plant-specific transcription factors including Teosinte branched1 (TB1) from maize, Cycloidea (CYC) from *Antirrhinum*, and Proliferating Cell Nuclear Antigen (PCNA) promoter-binding factors PCF1 and PCF2 from rice [23]. In addition, we show that H3K27me3 is enriched over the IR, implicating Polycomb Repressive Complex 2 (PRC2) in viral gene regulation. H3K27me3 is a hallmark of PRC2 activity. In plants and animals, PRC2 conditions metastable repression necessary to maintain differentiated cell states and has dynamic roles in response to developmental and environmental signals [24–26].

As a potential link between TCP24 and PRC2, we show that mutation of the TCP24 binding site (the CLE) in TGMV and CaLCuV DNA-A genome components results in a substantial reduction of H3K27me3 levels on viral chromatin in infected *Nicotiana benthamiana* and *Arabidopsis* plants. CLE mutation also delays and attenuates infection, with dramatic reductions in symptoms and viral DNA levels in systemically infected tissue. In contrast, in transient replication assays DNA-A components containing CLE mutations exhibit delayed replication but eventually accumulate dsDNA at levels similar to, or greater than, wild type DNA-A. However, viral ssDNA and CP mRNA levels are reduced, indicating roles for the CLE, TCP24, and PRC2 in *CP* promoter regulation. These findings suggest that TCP24 promotes repression of the *CP* promoter accompanied by H3K27me3 deposition, and recruits AL2 to derepress and drive *CP* expression.

## Results

### Isolation of cDNA encoding a TCP protein that interacts with a *CP* promoter sequence

In an earlier study, we employed a yeast one-hybrid screen to identify AtPPD2/TIFY4B, which interacts with AL2 and sequences within the IR of TGMV and CaLCuV [22,27]. To identify additional host factors, the screen was continued using the same target-reporter yeast strain (YM4271-TGMVCPactivator). The target-reporter construct, which is integrated into the genome of yeast strain YM4271, consists of four tandem copies of a 104 bp region of the TGMV *CP* promoter upstream of the yeast *HIS3* gene [22]. This region encompasses proximal elements involved in AL2-mediated promoter derepression and activation [9], including the CLE (Fig 1). The target-reporter strain was transformed with an expression library of *Arabidopsis* cDNAs fused to the GAL4 activation domain [28], and transformants expressing HIS3 were selected on synthetic complete (SC) medium lacking histidine and containing 3-aminotriazole (3-AT, 75 mM) to enforce HIS3 selection. Leucine was also omitted from the medium to ensure maintenance of cDNA-expressing plasmids. Putative positive transformants were identified, and expression plasmids were recovered and sequenced. One cDNA contained a complete coding region identical to AtTCP24 (At1g30210).

To confirm interaction between AtTCP24 and the *CP* promoter sequence, the target-reporter yeast strain was re-transformed with a plasmid expressing the cDNA fused to the Gal4 activation domain (pGAD-TCP24), or with empty plasmid vector (pGAD). Growth kinetics of the yeast strains in liquid SC medium lacking leucine and histidine with or without 3-AT (90 mM) were monitored over a 24-hour period. All strains grew on media lacking 3-AT, confirming the presence of each plasmid. Yeast strains containing pGAD-TCP24 also grew in the presence of 3-AT (S1 Fig), indicating positive interaction between TCP24 and the TGMV *CP* promoter. In contrast, yeast strains transformed with empty vector were unable to grow in media containing 3-AT.

### Sequence analysis of AtTCP24

Analysis of the AtTCP24 sequence revealed an open reading frame of 975 bp encoding a putative protein of 324 amino acids with a predicted molecular weight of 36.4 kDa. TCP proteins are plant-specific transcription factors, and multiple family members have been identified in several species [23]. For example, the genomes of *Arabidopsis*, tomato, and rice contain 24, 30, and 22 TCP genes, respectively [29–31]. The draft *N*. *benthamiana* genome available through SOL genomics suggests about 30 genes [32]. TCP proteins have an N-terminal, non-canonical basic helix-loop-helix domain, referred to as a TCP domain (~59 amino acids), that mediates DNA binding and protein-protein interactions [23]. In all species examined, TCPs can be divided into two classes (class I and class II) (S2 Fig), depending on differences in a highly conserved basic region within the TCP domain [29]. The basic region is necessary, but not sufficient, for DNA binding [33]. Moreover, DNA binding may involve dimerization, either through homo- or hetero-dimer formation, but only among members of the same class [34].

While the functions of all 24 *Arabidopsis* TCP proteins have yet to be described, in general class I TCPs (13 members) positively regulate cell growth and proliferation, while class II TCPs are negative regulators [35,36]. For example, a class II TCP (TCP24) is involved in repression of pre-replication complex genes [37]. Class II proteins can be further sub-divided into two well-defined groups, CINCINNATA (CIN, 8 members) and CYC/TB1 (3 members). AtTCP24 belongs to the class II CIN group. CIN-like TCP factors play key roles in leaf and flower development and can be regulated by environmental cues, including light and temperature as well as abiotic and pathogen stress [36]. Further, some class II CIN proteins, including TCP2, 3, 4, 10, and 24, are post-transcriptionally regulated by miR319 [38,39]. The mRNA sequences encoding tomato and *N*. *benthamiana* proteins that are most homologous to this subset also contain a putative target site for miR319 (S2 Fig) [30]. The conservation of TCP genes between species, and similar regulation, suggests conserved functions.

The studies reported here involve two host species, *Arabidopsis* and *N*. *benthamiana*, and two viruses. TGMV can infect *N*. *benthamiana*, while CaLCuV is able to infect both species. Because class II TCPs are present in both hosts, AtTCP24 will be referred to hereafter as TCP24.

### TCP24 binds the CLE within the TGMV and CaLCuV *CP* promoters

It is well established that class II TCP proteins bind the consensus sequence GTGGNCCC, which matches the CLE (GTGGTCCC) [18,34]. Electrophoretic mobility shift assays (EMSAs) were used to ask whether TCP24 binding to the *CP* promoter is mediated by this sequence. For these studies, full-length TCP24 was expressed in *E*. *coli* Rosetta cells as an N-terminal, glutathione-S-transferase fusion protein (GST-TCP24) and partially purified. For some experiments, TCP24 was also expressed as a six-histidine tagged fusion (6xHis-TCP24) (S3 Fig).

**Fig 2:**
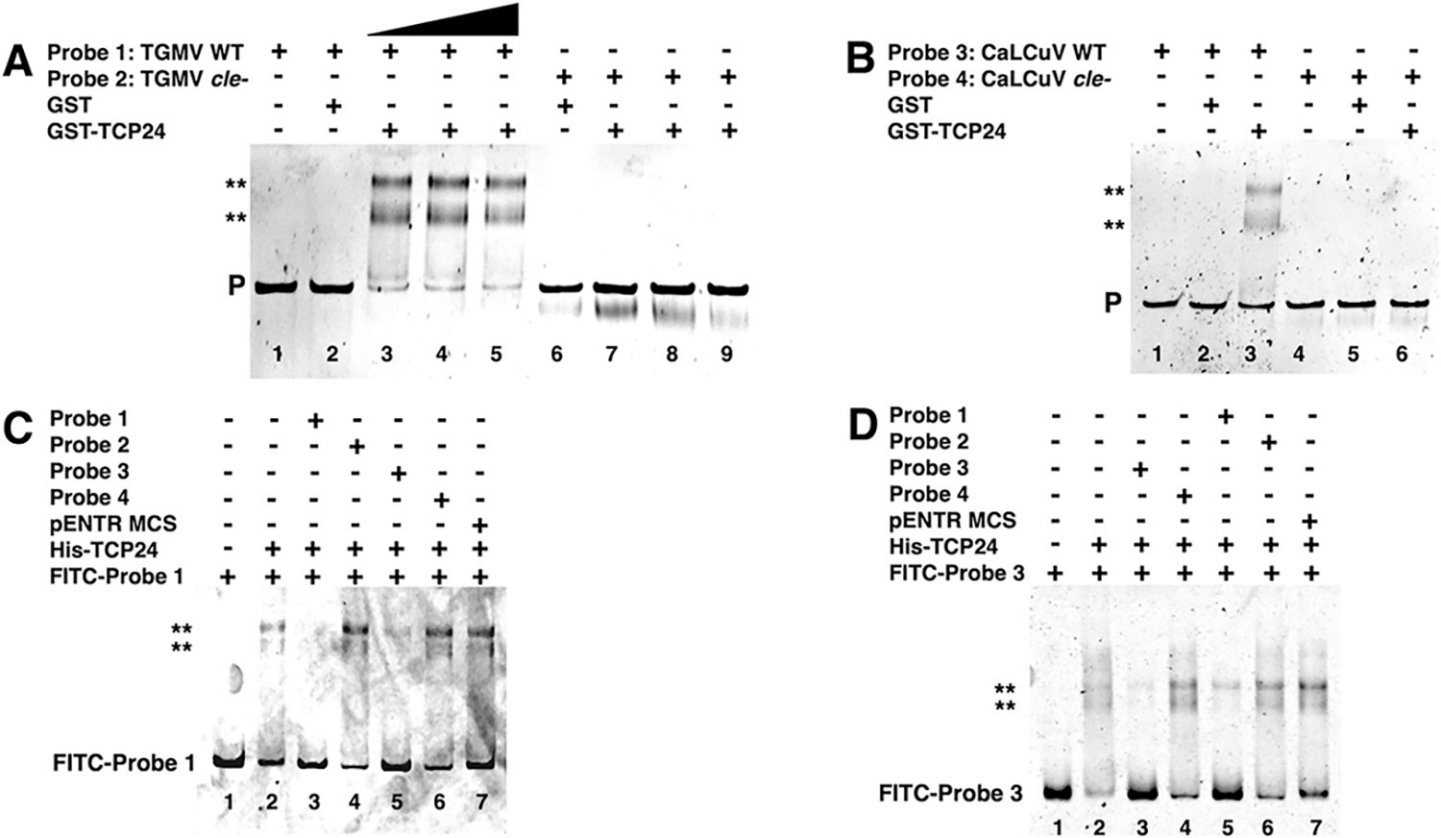
A class II TCP binding site (CLE) is necessary for efficient TCP24 binding to *CP* promoter sequences. GST-tagged or 6xHis-tagged TCP24 protein was isolated from *E*. *coli* Rosetta cells and purified as described in Methods. (A) Increasing volumes (6, 12, 18 μl) of purified GST-TCP24 protein were incubated with a TGMV *CP* promoter fragment containing a wild type (Probe 1) or mutated (Probe 2) CLE sequence. Protein-DNA complexes were separated on 2% agarose gel in 0.5X TB buffer, stained with ethidium bromide, and the positions of unbound probe (P) and TCP24 protein-probe DNA complexes (**) detected by UV illuminator. (B) GST-TCP24 was incubated with a CaLCuV *CP* promoter fragment containing a wild type (Probe 3) or mutated (Probe 4) CLE sequence. Protein-DNA complexes were separated and visualized as in (A). (C) Purified 6xHis-TCP24 protein (0.5 μg) was incubated with an FITC-labeled, wild type TGMV *CP* promoter fragment (Probe 1) in the presence (+) or absence (-) of a 50-fold molar excess of unlabeled competitor DNAs (Probes 1–4, as indicated). Protein-DNA complexes were separated on 6% polyacrylamide TBE gels and the positions of unbound probe and TCP24 protein-probe DNA complexes (**) detected with the iBright 1500 Imaging System. (D) Purified 6xHis-TCP24 protein (0.5 μg) was incubated with an FITC-labeled, wild type CaLCuV *CP* promoter fragment (Probe 3) in the presence (+) or absence (-) of a 50-fold molar excess of unlabeled competitor DNAs (Probes 1–4, as indicated). Protein-DNA complexes were separated and visualized as in (C). GST protein (A and B) and pENTR DNA (C and D) were negative controls. The images in (A) and (B) are representative of two independent experiments, and images in (C) and (D) of three independent experiments.

GST-TCP24 was incubated with a 299 bp DNA fragment (Probe 1, TGMV WT) encompassing the proximal elements of the TGMV *CP* promoter. The GST-TCP24 protein bound the *CP* promoter sequence as evidenced by a shift in the labeled probe (Fig 2A lanes 3–5). Two shifted products are apparent, which might represent TCP24 monomers and dimers bound to the probe DNA. As a negative control, partially purified GST was unable to bind the probe, demonstrating that binding was not due to the GST moiety (Fig 2A lane 2). In contrast, when GST-TCP24 was incubated with a similar probe containing a mutation of the class II TCP binding site (AGATCTTT; Probe 2, TGMV *cle*^-^), no binding was observed (Fig 2A lanes 7–9). Again, GST was unable to bind probe 2 (Fig 2A lane 6). Testing GST-TCP24 binding to the CaLCuV *CP* promoter employed a 290 bp DNA fragment analogous to that used for the TGMV promoter, with similar results. GST-TCP24 bound the CaLCuV *CP* promoter sequence as evidenced by two shifted products (Probe 3, CaLCuV WT), but not when it contained a mutation of the class II binding site (CTCGAGA; Probe 4, CaLCuV *cle*^-^) (Fig 2B lanes 3 and 6, respectively).

In addition, 6xHis-TCP24 binding to an FITC-labeled Probe 1 (TGMV WT) (Fig 2C lane 2) could be competed by excess (50-fold) unlabeled Probe 1 (TGMV WT) or Probe 3 (CaLCuV WT) (Fig 2C lanes 3 and 5, respectively), but not by excess unlabeled Probe 2 (TGMV *cle*^-^) or Probe 4 (CaLCuV *cle*^-^) (Fig 2C lanes 4 and 6, respectively). As a negative control, binding was not impacted by addition of an unlabeled, heterologous DNA fragment from pENTR MCS (Fig 2C lane 7). Similarly, binding of 6xHis-TCP24 to FITC-labeled Probe 3 (CaLCuV WT) (Fig 2D lane 2) could be competed by excess (50-fold) unlabeled Probe 3 (CaLCuV WT) or Probe 1 (TGMV WT) (Fig 2D lanes 3 and 5, respectively), but not by excess unlabeled Probe 4 (CaLCuV *cle*^-^) or Probe 2 (TGMV *cle*^-^) (Fig 2D lanes 4 and 6, respectively). As a negative control, binding was not impacted by addition of an unlabeled, heterologous DNA fragment from pENTR MCS (Fig 2D lane 7). From these results, we concluded that the CLE, identical to a class II TCP binding site, is necessary for efficient binding of TCP24 to both the TGMV and CaLCuV promoters.

As noted above, AL2 regulates the *CP* promoter through two separate regions in the genomes of TGMV and CaLCuV. One region is proximal to the promoter while the other is outside of the IR and contains a distal phloem repressor element located 1.2 to 1.5 kb upstream of the *CP* TSS [8]. Chromatin immunoprecipitation (ChIP) studies have shown that AL2 interacts with both regions *in vivo* [10]. However, 6xHis-TCP24 binding to an FITC-labeled TGMV *CP* promoter DNA (Probe 1, TGMV WT) was unaffected by a 50-fold excess of an unlabeled 105 bp DNA fragment containing the TGMV distal repressor element, or an unlabeled 144 bp DNA fragment containing the CaLCuV distal element (S4 Fig). Taken together, the results of EMSA experiments allow us to conclude that TCP24 binds proximal regions of the TGMV and CaLCuV *CP* promoters via the CLE but does not bind the distal repressor elements, which lack a CLE-like sequence.

### Analysis of begomovirus *CP* and *NSP* promoter regions reveals enrichment of class II TCP binding sites

Sequences within several geminivirus *CP* and *NSP* promoter regions contain a conserved late element (CLE) [18,40]. Although consensus binding sites for class I and class II TCP proteins exhibit some overlap (GGNCCCAC and GTGGNCCC, respectively) (34), the CLE sequence (GTGGTCCC) most closely matches the class II consensus and is necessary for efficient binding of TCP24, a class II TCP (Fig 2). We analyzed the *CP* and *NSP* promoter regions (400 to 500 bp upstream of translation start sites) from 43 bipartite begomoviruses, 4 monopartite begomoviruses, and two members of the genus *Curtovirus* for the presence of a class II binding motif. All viruses examined infect dicotyledonous plants. Among the bipartite begomoviruses analyzed, a two-fold enrichment for the TCP binding site sequence over that expected by chance (p-value < 2 ×10^−5^, Fisher’s exact test) was observed. Among bipartite virus genomes of both Old World and New World origin, 18 contained a sequence identical or similar to the class II TCP motif proximal to the *CP* coding sequence in either the viral or complementary strand. In 15 bipartite viruses such a sequence appeared in the B genome component proximal to the *NSP*/*BR1* coding region (S5 Fig). The motif was found in both genome components in 13 of these viruses, including TGMV and CaLCuV. In total, nearly half of the bipartite begomoviruses examined (20 of 43) contained at least one copy of the class II TCP motif. In addition, the motif appeared in three of the four monopartite begomoviruses, and both curtoviruses, examined. Interestingly, beet curly top virus (BCTV, a curtovirus) contains two CLE elements in the IR, and their deletion reduces transcription [41]. This suggests that curtovirus and begomovirus CLEs have overlapping functions even though the curtovirus L2 protein, which shares pathogenicity activities with AL2, cannot activate the viral *CP* promoter [17]. That a class II TCP motif frequently occurs in geminivirus genomes suggests that TCP binding is likely a common regulatory feature among dicot-infecting viruses.

### TCP24 interacts with CP promoter sequences in vivo

**Fig 3:**
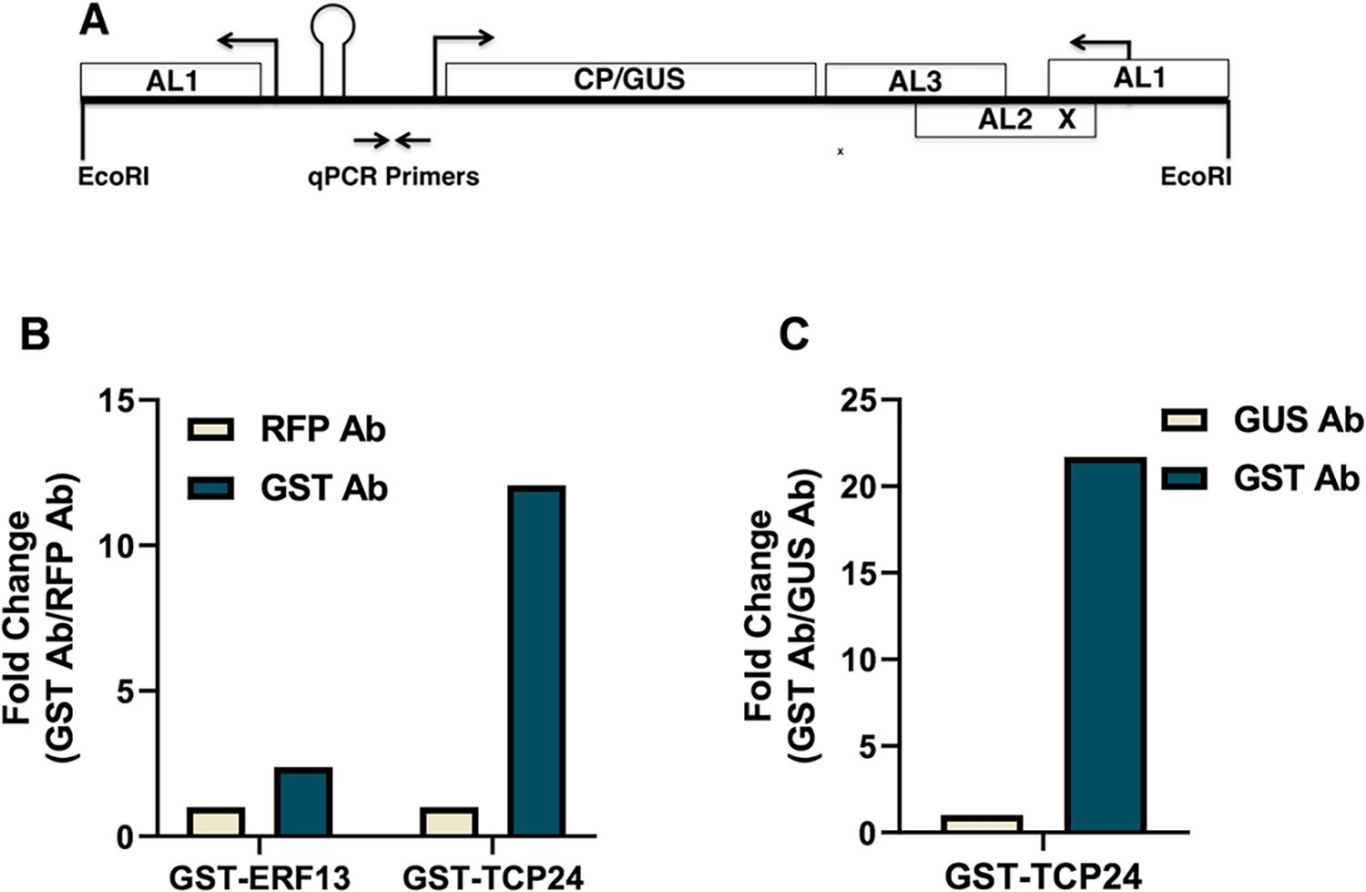
TCP24 interacts with the TGMV *CP* promoter *in vivo*. (A) The linear map represents the previously characterized TGMV A55M transgene [8]. Right angle arrows above the diagram indicate the positions of transcription start sites and arrows below the diagram indicate the positions of primers used for qPCR after ChIP. The CP coding region is replaced by GUS (CP/GUS) and AL2 is inactivated by mutation (AL2x). (B) Transgenic A55M *N*. *benthamiana* plants were infiltrated with *Agrobacterium* containing a TMV-based vector capable of expressing either GST-tagged TCP24 (GST-TCP24) or AtERF13 (GST-ERF13, negative control). Protein-DNA complexes were obtained by ChIP, and qPCR was performed on DNA in immune complexes using a primer set specific for the TGMV *CP* promoter. Fold change represents the difference in the amount of product detectable in qPCR reactions from samples immunoprecipitated using anti-GST antibody (GST Ab) relative to anti-RFP antibody (RFP Ab, negative control). (C) As in (B), except protein-DNA complexes from A55M plants expressing GST-TCP24 were captured by ChIP using anti-GST or anti-GUS (negative control).

TCP proteins are capable of localizing to the nucleus, which has been experimentally confirmed for some TCPs [29], and we confirmed for TCP24 (S6 Fig). We next employed ChIP to determine if TCP24 is able to interact with sequences known to mediate AL2-dependent regulation of the TGMV *CP* promoter in a chromatin context [8,9]. These experiments took advantage of previously characterized *N*. *benthamiana* lines carrying the A55M transgene [8], which consists of a full-length linear TGMV A genome in which the CP coding sequence has been replaced with the GUS reporter gene (Fig 3A). This construct also carries a mutation in the *AL2* gene and so is unable to produce a functional AL2 protein. Therefore, the *CP* promoter is inactive in these plants, but can be activated if AL2 is provided *in trans*. *Agrobacterium tumefaciens* cultures containing plasmids capable of delivering a tobacco mosaic virus (TMV)-based expression vector (TRBO) were used to infiltrate leaves of A55M plants [42]. TRBO vectors were designed to express GST-TCP24, or GST-ERF13 (*Arabidopsis* Ethylene Response Factor 13) as a negative control (S3 Fig). Based on our observation that most TCP24 localizes to the nucleus (S6 Fig), nuclear extracts were obtained two days post-infiltration. Protein-DNA complexes were isolated by immunoprecipitation using a rabbit-derived polyclonal primary antibody prepared against GST. As negative controls, immunoprecipitations were also performed using rabbit-derived polyclonal antibodies prepared against RFP or GUS. To assess enrichment following immunoprecipitations, qPCR was performed with primers to amplify a 109 bp fragment of the TGMV *CP* promoter (-22 to -131 bp upstream of the TSS) containing the CLE. Following expression of GST-TCP24, ChIP with anti-GST exhibited a ~12-fold enrichment of the 109 bp PCR fragment compared to anti-RFP (negative control) in the same samples (Fig 3B). In contrast, no enrichment was observed when immunoprecipitations were performed with anti-GST or anti-RFP following expression of GST-ERF13. In a separate experiment, immunoprecipitation with anti-GST yielded a ~22-fold enrichment of the 109 bp PCR product compared to anti-GUS (negative control) (Fig 3C). These results indicate that TCP24 specifically interacts with TGMV *CP* promoter proximal sequences *in vivo*.

An alternative explanation for ChIP results is that TCP24 interacts with the distal repressor element, and protein-DNA complexes detected by immunoprecipitation contain the entire TGMV genome due to incomplete chromatin fragmentation. However, in control experiments, it was only possible to PCR-amplify a 2150 bp fragment containing the *CP* promoter and the GUS coding region, which lies between it and the distal repressor, from A55M DNA extracts obtained before, but not after, sonication (S7 Fig). Thus, the sonication step in the ChIP protocol (shear size ~600 bp) was sufficient to uncouple the two regions of the TGMV genome. This supports our interpretation that TCP24 interacts *in vivo* with the CLE motif proximal to the *CP* promoter.

### TCP24 interacts with AL2

A goal of the one-hybrid screen was to identify cellular proteins that interact with *CP* promoter sequences, and also with viral AL2 protein. Both TCP24 and AL2 self-interact [34,43], and possibly because of this it proved challenging to test interaction between these proteins in the yeast two-hybrid system. However, TCP24 and AL2 self-interaction, and TCP24-AL2 interaction, could be detected by protein-protein gel blot (Far Western) analysis. These experiments employed 6xHis-tagged TCP24, 6xHis-TGMV AL2, and 6xHis-GFP (negative control), with GST-TCP24 as probe.

**Fig 4:**
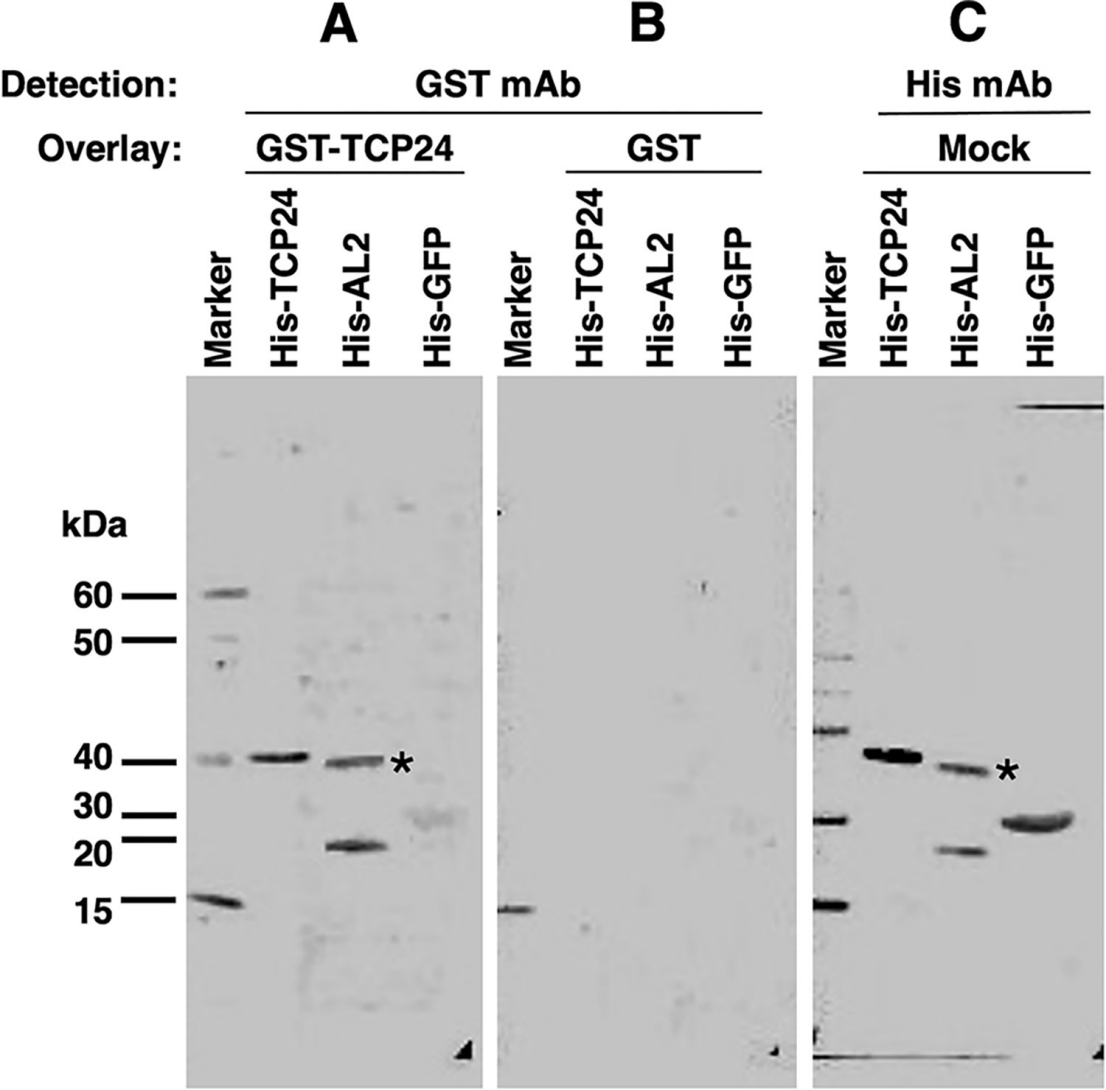
TCP24 interacts with TGMV AL2 protein. Protein-protein (Far Western) gel blots are shown. Samples of 6xHis-TCP24, 6xHis-AL2, and 6xHis-GFP were resolved on 4–20% protein gels and gel blots were overlayed with a soluble fraction from *E*. *coli* expressing either (A) GST-TCP24, (B) GST, or (C) buffer only (Mock). Membranes overlayed with GST-TCP24 or GST were incubated with an anti-GST monoclonal antibody. The mock was incubated with an anti-6xHis monoclonal antibody. The position of protein standards is shown on the left and asterisks indicate the AL2 protein dimer. The images are representative results from 9 independent experiments.

When blots containing 6xHis-TCP24, -AL2, and -GFP test proteins were incubated with GST-TCP24 (primary probe, overlay) followed by GST antibody (secondary probe), no signal was detected in the GFP negative control. However, signals corresponding to 6xHis-TCP24 and 6xHis-AL2 were observed, indicating GST-TCP24:6xHis-TCP24 and GST-TCP24:6xHis-AL2 interaction (Fig 4A). Interestingly, both mono- and dimeric forms of AL2, which are routinely observed following gel electrophoresis [43], appeared to interact with TCP24. In contrast, when GST alone was overlayed as primary probe over an identical protein blot, no signal was observed for any of the test proteins (Fig 4B), confirming that interactions were not due to the GST moiety. Expression of all 6xHis-tagged test proteins was confirmed when an identical protein blot was probed with His antibody (mock overlay) (Fig 4C). Thus, these experiments verified previously reported self-interactions of AL2 and TCP proteins, and in addition provided evidence for direct TCP24-AL2 interaction.

**Fig 5:**
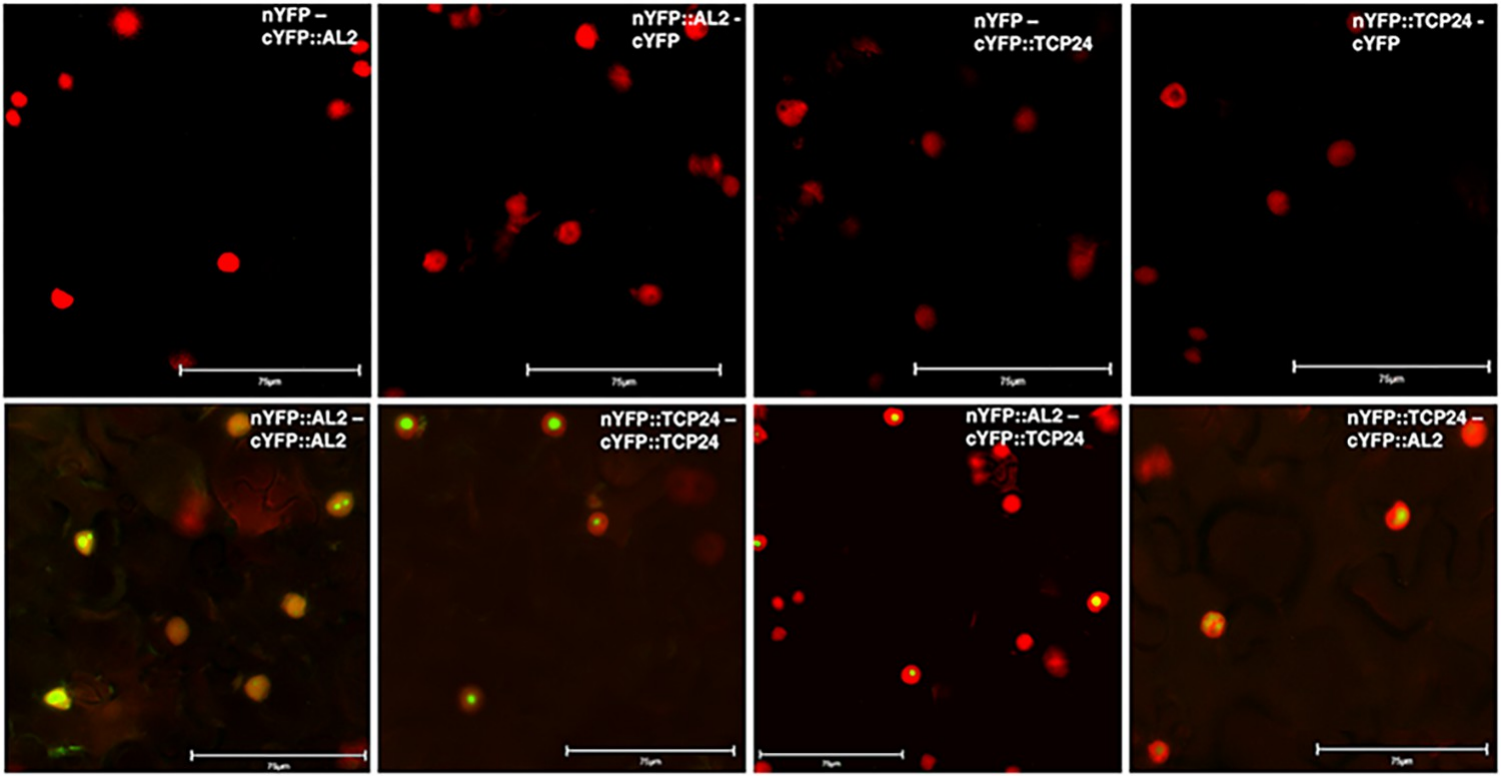
Bimolecular Fluorescence Complementation (BiFC) analysis of TCP24-AL2 complexes in *N*. *benthamiana* epidermal cells. Constructs expressing full length AL2 protein or TCP24 fused to the N- or C-terminal portion of YFP were delivered to *N*. *benthamiana* leaves by agroinfiltration. Images were examined for fluorescence indicative of interaction with a 40x objective using FITC (eGFP signal) and Rhodamine (RFP-Histone H4 signal) filter sets. Photographs represent merged images from the two filter sets. H4-RFP localizes to the nucleus and is identified by red fluorescence, while reconstituted YFP is identified by green fluorescence. Protein combinations are indicated on each image, with the protein listed first fused to the N-terminal portion of YFP, and the second fused to the C-terminal portion. Controls expressed either nYFP or cYFP. Panels including AL2-AL2 and TCP24-TCP24 are tests of self-interactions. Scale bars indicate 75 μm. The images are representative of results from three independent experiments.

Bimolecular fluorescence complementation (BiFC) was employed as a means of testing TCP24-AL2 interaction *in vivo*. To do this, TCP24 and TGMV AL2 were fused to the N- or C-terminal portions of YFP, and constructs were introduced into epidermal cells of *N*. *benthamiana* plants by agroinfiltration. Protein association reconstitutes YFP, resulting in fluorescence which indicates interaction and where interacting proteins accumulate in the cell. To enhance detection, a construct containing an inter-peptide linker sequence coding for six alanine residues was inserted between the YFP and TCP24 coding sequences [44]. Analysis by fluorescence microscopy further confirmed TCP24 and AL2 self-interactions and revealed TCP24-AL2 interaction within the nucleus (Fig 5), based on an overlapping signal with a nuclear marker (histone H4-RFP) [45], which exhibits red fluorescence. These results demonstrate that AL2 and TCP24 are able to interact *in vivo*, corroborating interactions observed by Far Western analysis.

Co-immunoprecipitation (Co-IP) experiments were performed as an additional verification of *in vivo* TCP24-AL2 interaction. In these experiments, *Agrobacterium* containing plasmids to deliver TRBO vectors to express FLAG-AL2, GST-AL2, GST-TCP24, GST (control), or TRBO (empty vector) were agroinfiltrated to *N*. *benthamiana* plants. Cultures were mixed prior to infiltration so that all GST-tagged proteins were separately co-expressed with FLAG-AL2. Tissue from infiltration zones was harvested after 48 hours and fixed with formaldehyde, after which nuclei were isolated and sonicated. Soluble fractions were used for Co-IP reactions.

**Fig 6:**
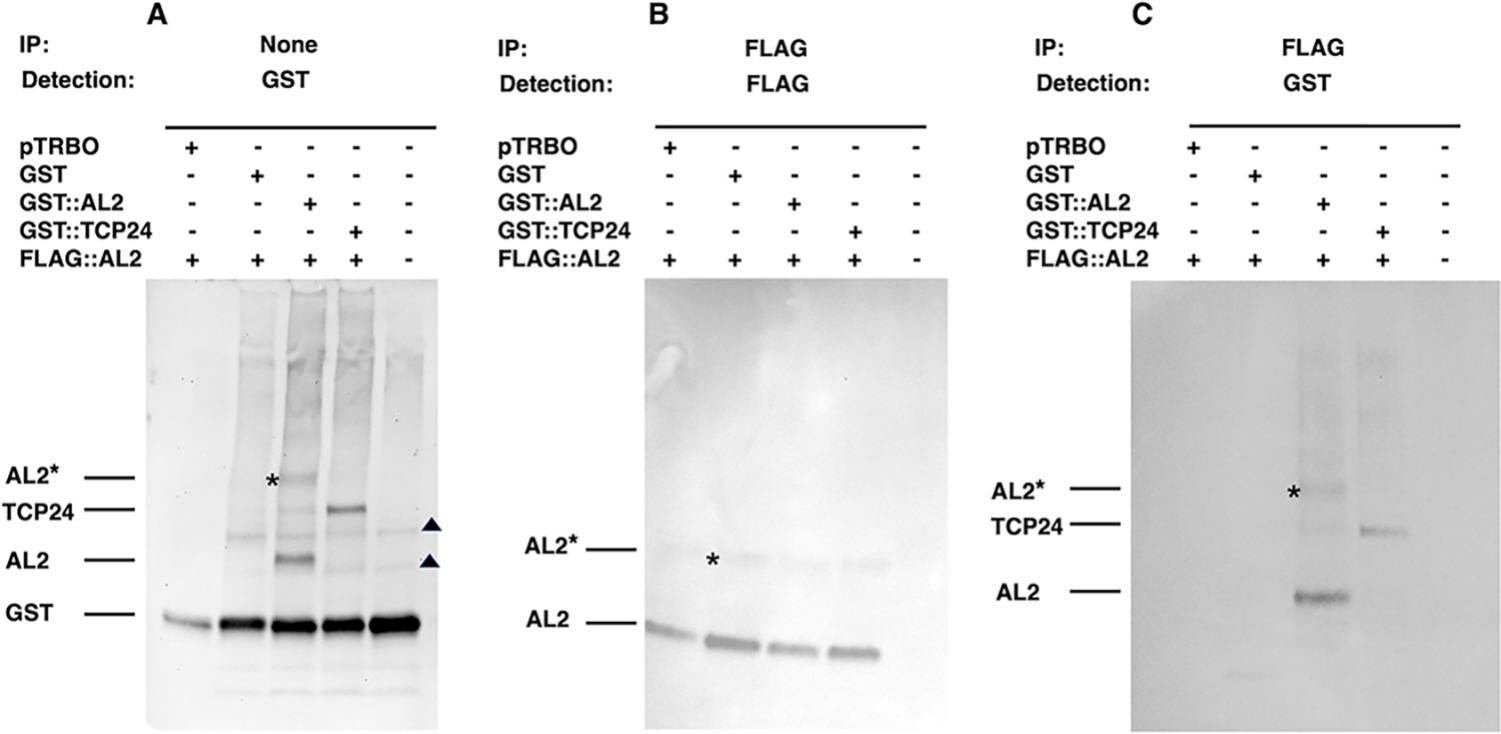
TCP24 co-immunoprecipitates with TGMV AL2 protein. Constructs to express FLAG-AL2, GST-AL2, GST-TCP24, GST (control), or TRBO (empty vector) were agroinfiltrated to *N*. *benthamiana* leaves. Cultures were mixed prior to infiltration so that all GST-tagged proteins were separately co-expressed with FLAG-AL2. Tissues were obtained from infiltration zones and extracts used for Co-IP reactions. Protein (Western) gel blots are shown. Rightmost lanes in each blot contained protein from non-infiltrated tissue. (A) Protein blot probed with GST antibody. (B) IP with FLAG antibody followed by protein blot probed anti-FLAG. (C) IP with FLAG antibody followed by protein blot probed with anti-GST. Asterisks indicate dimeric AL2*. Triangles indicate cross-reacting (background) proteins.

When co-expressed with FLAG-AL2, all GST-tagged proteins were detected by protein gel blot (Western blot) probed with GST antibody, confirming expression of these proteins (Fig 6A, input). Here, GST signal was observed in all lanes due to the presence of endogenous GST and again, both mono- and dimeric forms of GST-AL2 were apparent. Following IP with anti-FLAG, immunoprecipitates were examined by protein blot probed with the same antibody. Mono- and dimeric forms of AL2 were observed in all cases, confirming expression of FLAG-AL2 and immunocapture by anti-FLAG antibody (Fig 6B). Finally, after IP with anti-FLAG and protein blot with anti-GST, immunoprecipitates containing GST-AL2 and GST-TCP24 were observed (Fig 6C), indicating FLAG-AL2:GST-AL2 (both mono- and dimeric forms) as well as FLAG-AL2:GST-TCP24 interaction. By contrast, FLAG antibody failed to bring down GST. These results confirm AL2 self-interaction as well as TCP24-AL2 interaction, and validate results from Far Western and BiFC analysis.

### CLE mutation decreases the ability of TGMV and CaLCuV to initiate systemic infection

Based on results showing that TCP24 binding to the *CP* promoter depends on the CLE (Fig 2), and that TCP24 interacts with AL2 (Figs 4, 5 and 6), we hypothesized that mutation of the putative class II TCP binding site (CLE) would lead to loss or impairment of infection. The underlying rationale is that absence of TCP24 binding would result in dysregulation of CP expression, resulting in decreased virus accumulation and/or spread. DNA clones with tandemly repeated copies of the TGMV (1.75-mer) or CaLCuV (1.5-mer) DNA-A genome components were constructed that contained a single IR, and therefore a single copy, of the wild type CLE (GTGGTCCC). A similar series of DNA-A clones containing mutations in the CLE of TGMV (AGATCTTT) or CaLCuV (TCTCGAGT) were also generated. *N*. *benthamiana* plants were agroinoculated with wild type or mutant (*cle-*) DNA-A components, along with wild type cognate DNA-B components, and assessed for the appearance of symptoms. In independent experiments, each with two independent replicates, the average time to the appearance of systemic symptoms (mean latent period) typical of wild type TGMV infection was between 7–7.5 days, with 89–100% of plants becoming infected (Table 1). In contrast, symptoms were delayed and appeared between 10 to 13 days in plants inoculated with the TGMV *cle*- mutant virus, with 65–92% of plants becoming infected. Symptoms were also less severe in plants infected with the TGMV *cle*- mutant virus (S8 Fig).

**Table 1:** Infectivity of CaLCuV and TGMV wild type and *cle-* mutant viruses in *N*. *benthamiana*.

Virus^a^	EXPT. #1		EXPT #2	
	Plants Infected^b^	MLP^c^	Plants Infected^b^	MLP^c^
WT TGMV	11/12 (92%)	7.45 ± 0.39	16/18 (89%)	7.44 ± 0.43
WT TGMV	12/12 (100%)	7.00 ± 0.37	17/17 (100%)	7.47 ± 0.39
*cle-* TGMV	11/12 (92%)	10.36 ± 1.26*	11/17 (65%)	10.18 ± 0.96*
*cle-* TGMV	11/12 (92%)	13.00 ± 1.24*	ND	ND
WT CaLCuV	10/12 (83%)	14.90 ± 2.07	14/18 (78%)	7.00 ± 0.28
WT CaLCuV	9/12 (75%)	13.22 ± 2.10	12/17 (71%)	7.42 ± 0.54
*cle-* CaLCuV	0/12 (0%)	AS	3/18 (17%)	MS
*cle-* CaLCuV	0/12 (0%)	AS	3/18 (17%)	MS

^a^Two independent clones were used for each experiment

^b^The number of plants infected/number of plants inoculated is shown

^c^Mean latent period is shown in days ± standard error of the mean

*Significant difference at p < 0.01

AS: Plants inoculated were asymptomatic

MS: Plants exhibited very mild symptoms

ND: Not determined

In *N*. *benthamiana* plants agroinoculated with CaLCuV, a mean latent period typical for a wild type infection ranged from 13–15 days in one experiment, with 75–83% of plants becoming infected, and 7–7.5 days in a second experiment with 71–78% of plants becoming infected. Interestingly, plants inoculated with the CaLCuV *cle*- mutant virus did not develop detectable symptoms in one experiment, and only 3 of 18 plants exhibited mild symptoms in each replicate of the second experiment (Table 1) (S8 Fig). Due to extreme attenuation or complete absence of symptoms, it was not possible to determine a mean latent period for CaLCuV *cle-* infections, even though viral DNA could be detected in some asymptomatic plants (see below). We concluded that in *N*. *benthamiana*, mutation of the CLE in TGMV DNA-A results in a significant delay (3–6 days) and attenuation of symptoms, whereas CLE mutation in CaLCuV DNA-A results in very severe attenuation or absence of disease symptoms.

**Fig 7:**
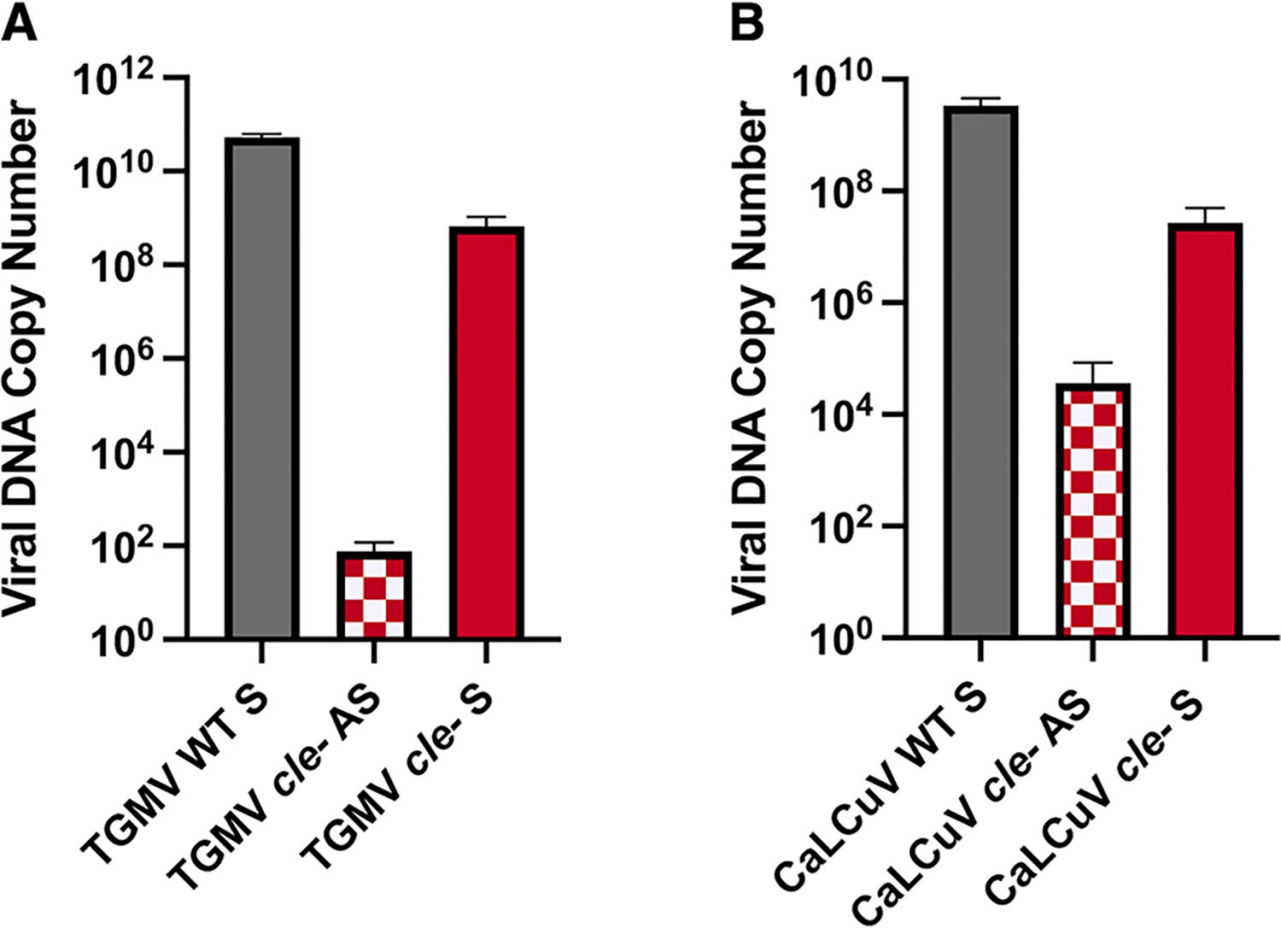
Viral DNA loads in *N*. *benthamiana* plants infected with wild type or *cle-* mutant TGMV or CaLCuV. Total DNA was isolated from systemic tissue infected with wild type or *cle-* mutant viruses and qPCR performed using primers specific for the (A) TGMV or (B) CaLCuV CP ORF. The bars illustrate the average amount of viral DNA (log^10^ scale) present in systemic leaf tissue away from the site of inoculation in individual plants that were symptomatic (S) or asymptomatic (AS) following inoculation with wild type (WT) or *cle-* mutant viruses. Viral DNA copy numbers were calculated by comparison to a standard curve. Statistical differences between pairs were determined by ANOVA followed by a Tukey-Kramer post-hoc comparison. Individual values for viral DNA amounts (ng) and number of copies of the viral genome are shown in S1 and S2 Tables.

To determine whether the delay and attenuation of symptoms observed was a consequence of reduced viral DNA accumulation in plants infected with the *cle-* mutant viruses, total DNA was isolated from systemically infected tissue 15 days post-inoculation (dpi) and viral DNA levels were quantified by qPCR. From six randomly selected plants infected with wild type TGMV in Experiment 1 (Table 1), an average of 5.4 x 10^10^ copies of the viral genome (150 ng viral DNA) per 1 μg tissue was observed (S1 Table) (Fig 7A). In contrast, symptomatic tissue from six randomly selected plants infected with TGMV *cle-* (Experiment 1) contained up to 125-fold less viral DNA (6.7 x 10^8^ copies of the viral genome), while asymptomatic tissue contained very little viral DNA (7.5 x 10^1^ copies of the viral genome). Total amounts of DNA in plants infected with wild type CaLCuV were somewhat less than observed with TGMV, however, results were similar (S2 Table) (Fig 7B). Plants infected with wild type CaLCuV contained an average of 3.4 x 10^9^ copies of the viral genome (9.4 ng viral DNA), with significantly lower levels detectable in the mildly symptomatic tissue from plants infected with the CaLCuV *cle-* mutant virus (3.2 x 10^7^ copies of the viral genome). Asymptomatic tissue from plants infected with CaLCuV *cle-* contained considerably less viral DNA (3.5 x 10^4^ copies of the viral genome) (S2 Table) (Fig 7B).

**Fig 8:**
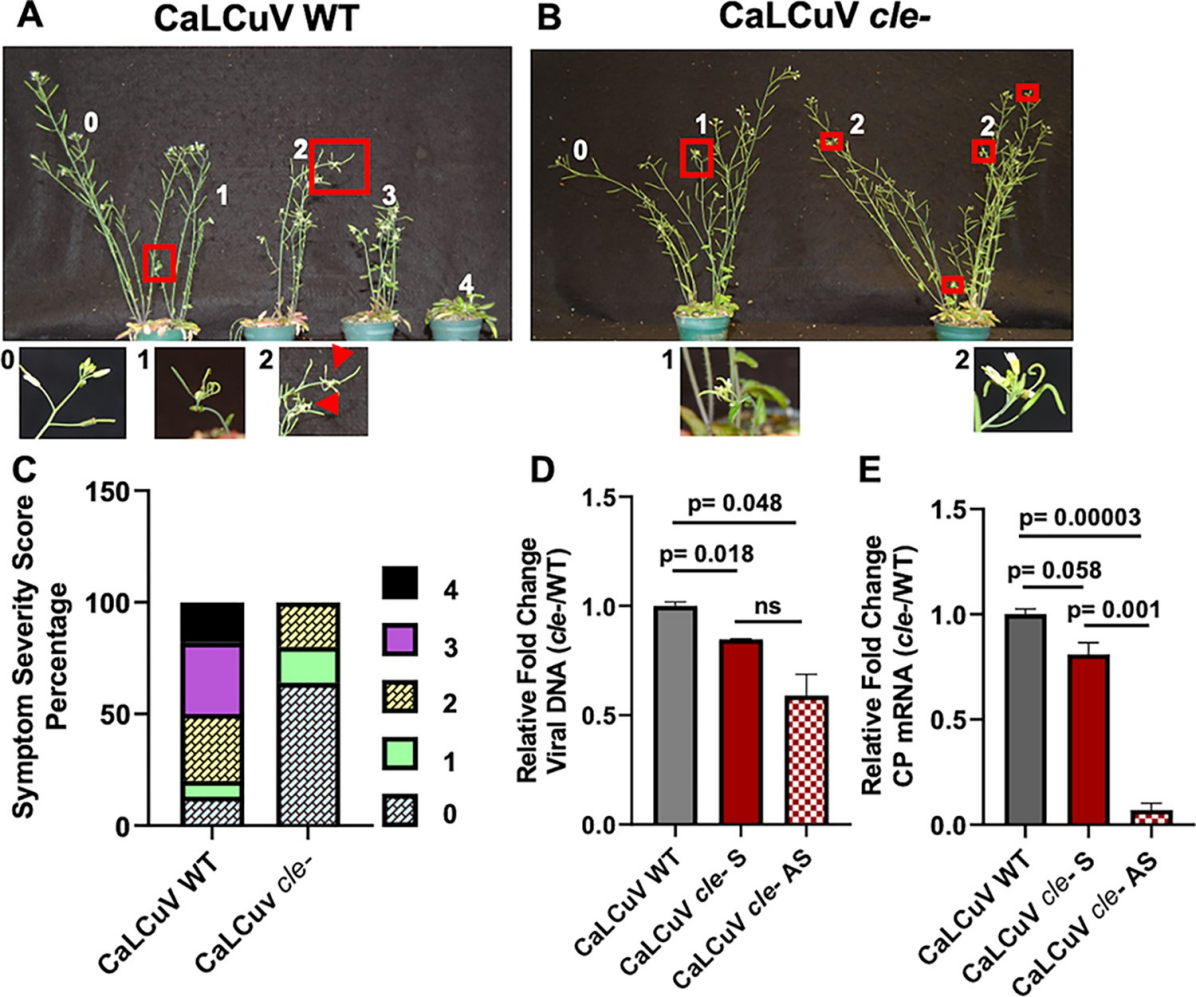
Quantitation of symptom severity, viral DNA, and CP mRNA in systemically infected *Arabidopsis*. (A) Representative photograph illustrating symptom severity range for wild type (WT) CaLCuV in *Arabidopsis* (ecotype Col-0). Photos were taken at 22 dpi. Red boxes highlight deformed bolts and numbers indicate severity scores: Asymptomatic = 0; mild deformation of floral heads and siliques on a single bolt = 1; mild deformation of floral heads and siliques on multiple bolts = 2; moderate deformation of floral heads and siliques on multiple bolts coupled with mild to moderate stunting = 3; severe deformation of floral heads and siliques coupled with severe stunting = 4. (B) Representative photograph of CaLCuV *cle-* symptom severity range. (C) Quantitation of symptom severity scores for both CaLCuV WT (n = 44 plants) and CaLCuV *cle-* (n = 45 plants). Percentage of plants with each score is illustrated. (D) Viral DNA levels. Total viral DNA was quantified by qPCR using a primer set specific for the CP coding region and normalized to 18S ribosomal DNA. Wild type CaLCuV was set to a value of 1.0. Significance was calculated using an unpaired Student’s *t*-test. Bars indicate standard error of the mean of three biological replicates. S, symptomatic tissue, AS, asymptomatic tissue. (E) CP mRNA levels. Total RNA was extracted from the same samples as (D) and CP mRNA measured by RT-qPCR using primers specific for the CP coding region. Viral RNA was normalized to total viral DNA as measured by qPCR, then normalized to PP2A.

As TGMV does not infect *Arabidopsis*, CaLCuV was used to repeat experiments in this host. To observe a range of symptom development, plants were infected under conditions that normally produce relatively mild symptoms on reproductive shoots (bolts). In two independent experiments, symptoms on *Arabidopsis* plants agroinoculated with *cle*- mutant CaLCuV were highly attenuated relative to the wild type virus, with a mean latent period of 14.2 days compared to 10.8 days for wild type. The impact of the mutation on infectivity was further evaluated using a rating scale for symptom severity, with 0 assigned to asymptomatic plants and 4 to plants displaying severe deformation of siliques and flowers accompanied by severe stunting (Table 2) (Fig 8A). Under the conditions employed, the majority of plants inoculated with wild type CaLCuV exhibited symptoms (38/44 plants, 86%) with most infected plants showing mild to moderate silique and floral deformation on multiple bolts accompanied by mild to moderate stunting (average severity score = 2.71). In contrast, many fewer plants inoculated with CaLCuV *cle-* exhibited symptoms (16/45, 36%), and plants that did showed mild to moderate silique and floral deformation with no stunting (average severity score = 1.56) (Fig 8B). None of the *Arabidopsis* plants inoculated with CaLCuV *cle-* exhibited symptoms with a severity rating greater than 2, whereas 18% of plants infected with wild type CaLCuV exhibited severe symptoms (severity score = 4) (Fig 8C and Table 2).

**Table 2:** Infectivity of CaLCuV wild type and *cle-* mutant viruses in *Arabidopsis*.

Virus	CaLCuV WT	CaLCuV cle-	p value^d^
#Symptomatic/Inoculated	38/44 (86%)	16/45 (36%)	NA
Range of symptom scores^a^	0–4	0–2	NA
Average symptom score^b^	2.71 ± 0.14	1.56 ± 0.13	3 x 10^−7^
Mean Latent Period^c^	10.80 ± 0.80	14.16 ± 0.37	9 x 10^−3^

^a^Symptom severity score is based on the scale: 0—no symptoms; 1—mild silique/floral deformation on a single bolt, no stunting; 2—mild to moderate silique/floral deformations on multiple bolts, no stunting; 3—moderate silique/floral deformations on multiple bolts, mild to moderate stunting; 4—major deformations of silique and flowers, severe stunting or no growth

^b^Average symptom severity score is reported ± the standard error of the mean

^c^Mean latent period is shown in days ± standard error of the mean

^d^ Significance of mean latent period and average symptomatic scores was confirmed by a two-tailed Student’s *t*-test. NA = not applicable.

Viral DNA could be detected at 22 dpi by qPCR in extracts from both symptomatic and asymptomatic plants inoculated with wild type or *cle*- CaLCuV. Viral DNA loads were significantly lower in symptomatic tissues infected with the *cle*- mutant compared to wild type virus, with the largest reduction seen in asymptomatic plants (Fig 8D). However, differences were not as great as those observed in *N*. *benthamiana*, possibly because different tissues were analyzed (*N*. *benthamiana* leaves vs. *Arabidopsis* bolts). Nevertheless, analysis of infection phenotypes in *N*. *benthamiana* and *Arabidopsis* plants clearly demonstrates that mutation of the CLE in CaLCuV and TGMV DNA-A components attenuates infection.

### Transcript levels of several TCPs are altered by CaLCuV infection

**Fig 9:**
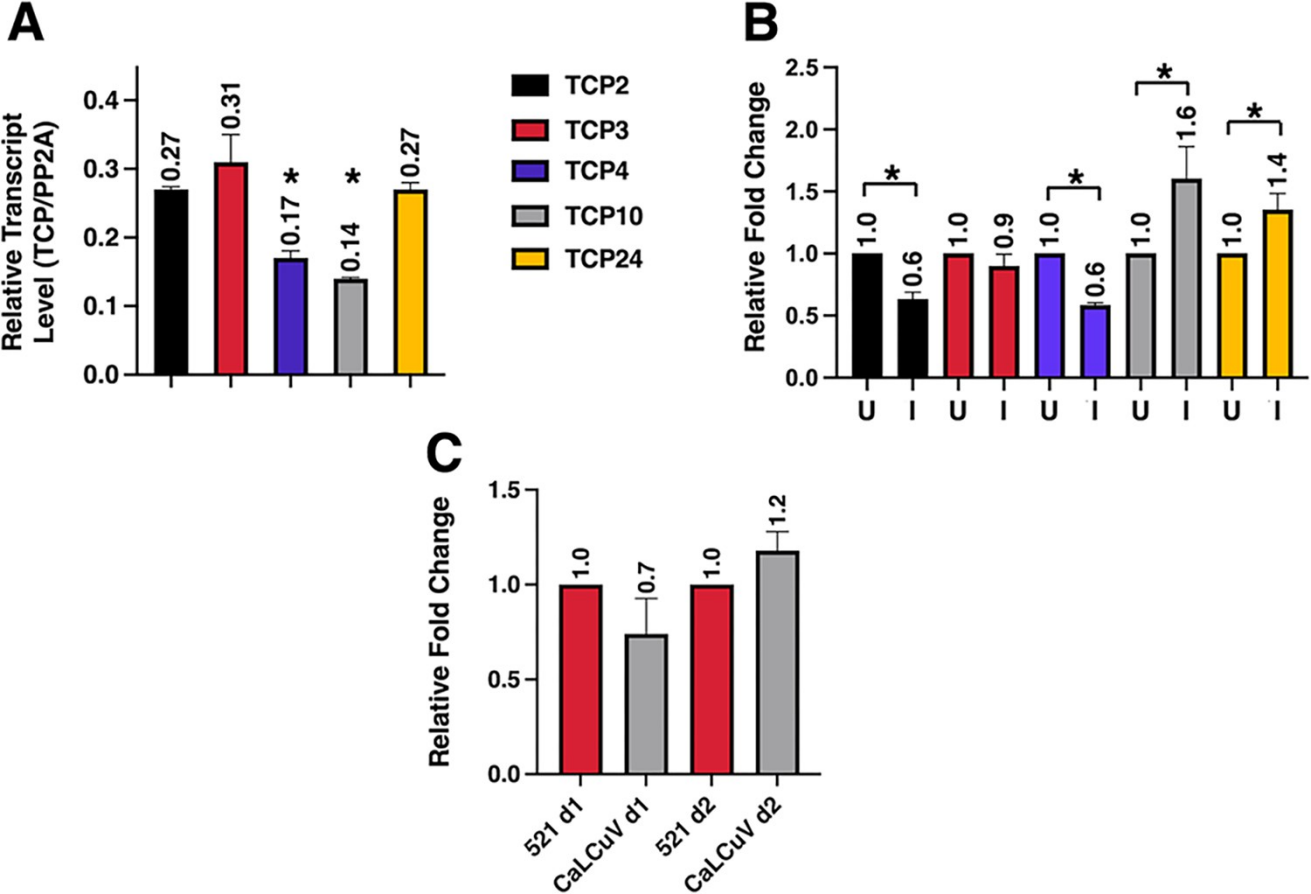
Multiple class II TCP transcript levels are altered by CaLCuV infection. (A) Total RNA was obtained from uninfected *Arabidopsis* plants, and TCP2, 3, 4, 10, and 24 transcript levels were determined by RT-qPCR. Transcript levels were normalized to PP2A. (B) Total RNA was obtained from *Arabidopsis* plants 22 days after agroinoculation with WT CaLCuV DNA-A and DNA-B. Transcript levels in infected plants (I) were normalized to PP2A and compared to levels in uninfected plants (U). (C) *Arabidopsis* seedlings were vacuum infiltrated with *Agrobacterium* cultures to deliver CaLCuV DNA-A or empty Ti plasmid vector (pMON521). Total RNA was isolated one- and two-days post-infiltration and TCP24 transcript levels determined by RT-qPCR, and normalized to PP2A. Experiments included at least three replicates, and bars indicate standard error. Asterisks indicate significant differences (p < 0.05) as determined by Students *t*-test.

Because mutation of the TCP24 binding site (the CLE) in CaLCuV impacts disease symptoms and viral DNA levels in *Arabidopsis*, we wondered if TCP24 expression might be altered in infected plants. Since class II TCPs bind the same sequence and could be more or less functionally redundant, changes in steady-state transcript levels of *Arabidopsis* TCP24 and the four TCPs to which it is most closely related (i.e., TCP2, TCP3, TCP4, TCP10) were determined by RT-qPCR. Extracts were obtained 22 dpi from bolt tissues of uninfected, mock inoculated plants and plants infected with wild type CaLCuV, and analyses were performed using five different primers sets designed to be specific for each individual TCP. Transcript levels were normalized to PP2A. This analysis revealed that in uninfected *Arabidopsis*, TCP4 and TCP10 are expressed at lower levels than TCP2, TCP3, and TCP24 (Fig 9A). Further, the transcript levels of some TCPs were significantly altered by CaLCuV infection. TCP2 and TCP4 levels were decreased (~1.6-fold), while TCP10 and TCP24 transcript levels were upregulated (~1.6- and 1.4-fold, respectively) in infected compared to uninfected plants (Fig 9B). These results suggest that several TCPs, and importantly TCP24, are responsive to geminivirus infection.

TCP24 transcript levels were also examined in transient infection experiments. Here, *Arabidopsis* seedlings were vacuum infiltrated with *Agrobacterium* cultures to deliver wild CaLCuV DNA-A, as previously described [44]. Negative controls consisted of seedlings infiltrated with cultures containing the empty Ti plasmid vector (pMON521). RNA was isolated one- and two-days post-infiltration, and RT-qPCR was performed using primers specific for TCP24. Compared to empty vector controls, TCP24 transcript levels in infected plants decreased after day 1 but recovered and were somewhat increased by day 2 (Fig 9C), further suggesting that TCP24 responds to geminivirus infection.

### CLE mutation does not diminish overall viral DNA accumulation in transient assays

**Fig 10:**
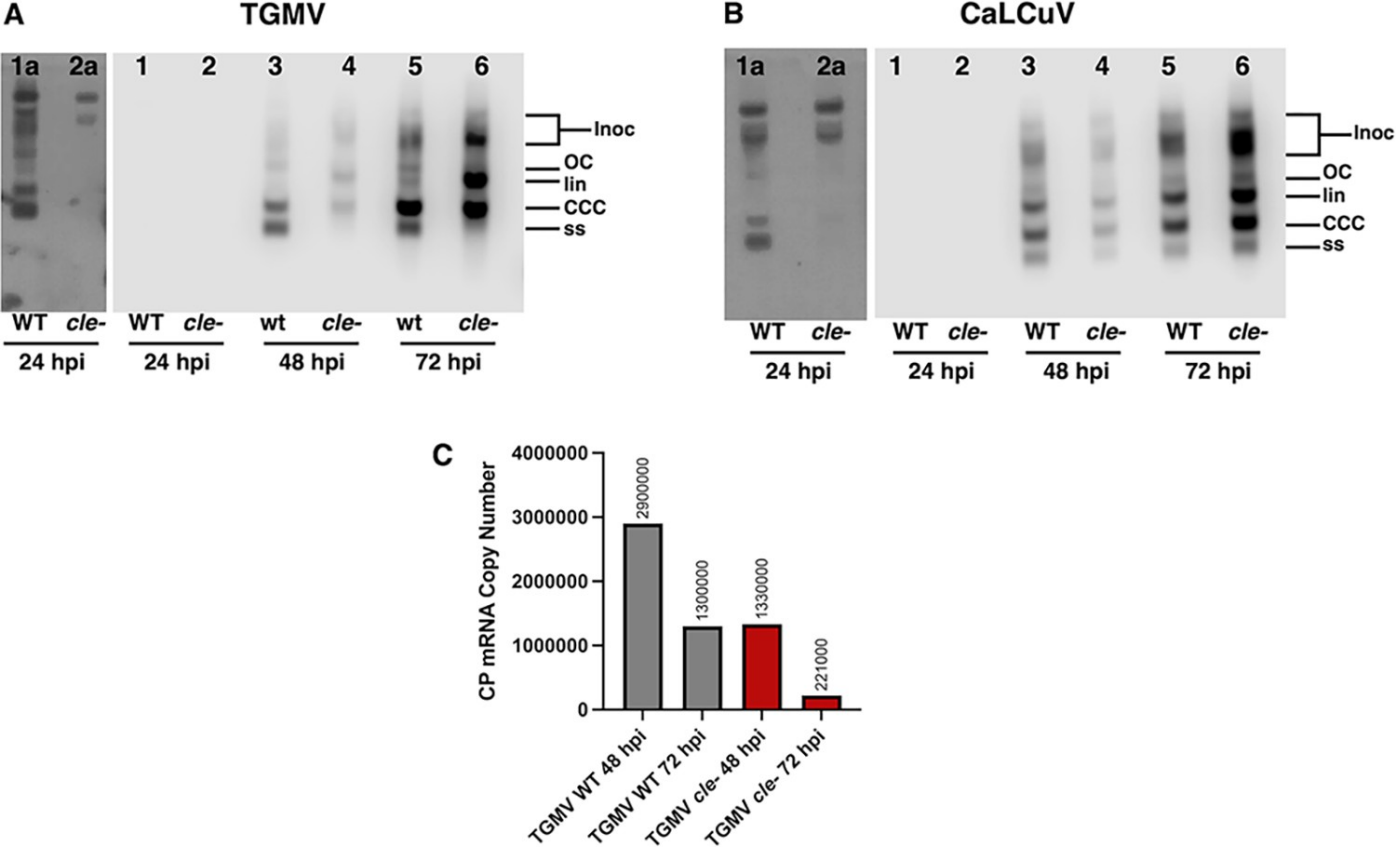
Time course of viral DNA and CP mRNA accumulation in *N*. *benthamiana* plants infiltrated with wild type or *cle-* mutant TGMV or CaLCuV. Leaves from *N*. *benthamiana* plants were infiltrated with *Agrobacterium* cultures containing wild type (WT) or *cle-* TGMV or CaLCuV DNA-A and total DNA isolated 24- to 72 hours post-inoculation (hpi). Images shown are scans of Southern blots hybridized to probes specific for TGMV (A) or CaLCuV (B) DNA-A as detected by chemiluminescence (Lanes 1–6). Lanes 1a and 2a are images of longer exposures to detect viral DNA at 24 hpi. Covalently closed circular (ccc), open circular (oc) and linear (lin) double-stranded DNA forms, as well as single-stranded (ss) viral DNA forms, are indicated. Residual inoculum DNA (Inoc) is also indicated. Relative levels and absolute chemiluminescence units for each viral DNA form are given in S3 Table. (C) Samples were isolated from the same tissue as the DNA shown above (A and B) and CP mRNA copy number was determined by RT-qPCR using a standard curve. The graph illustrates the number of copies of TGMV CP mRNA detected in total RNA isolated 48 and 72-hpi from leaves of *N*. *benthamiana* plants infused with *Agrobacterium* cultures containing wild type (WT) or *cle-* mutant TGMV DNA A. Levels of CP mRNA were adjusted for dsDNA levels and data are shown in S4 Table.

Because the CLE lies within the IR adjacent to the viral origin of replication, reduced levels of viral DNA in plants infected with *cle-* mutant viruses could either be the result of a direct effect on viral DNA replication, or an indirect effect of aberrant gene expression leading to reduced systemic spread. To distinguish between these possibilities, viral DNA accumulation was examined over time in a transient assay. *N*. *benthamiana* leaves were infiltrated with *Agrobacterium* cultures to deliver wild type or *cle-* DNA-A. DNA-B was omitted from these experiments in order to minimize cell-to-cell spread. Total DNA was isolated from infiltration zones 24-, 48- and 72-hours post-inoculation (hpi), and viral DNA levels were assessed by DNA gel blot hybridization. Signals were quantified by chemiluminescence. With wild type TGMV, replicating DNA could be detected by 24 hpi upon extended exposure (Fig 10A lane 1a). Visible were open circular (OC), linear (Lin), and covalently closed circular (CCC) dsDNA forms, as well as circular ssDNA (ss). Levels of all viral DNA forms increased after 48 and 72 hpi (Fig 10A lanes 3 and 5) (S3 Table). Similar replication kinetics were previously observed following transfection of protoplasts derived from *Nicotiana tabacum* suspension culture [46]. By comparison, replicating viral DNA was not detected after 24 hpi in leaves infiltrated with the TGMV *cle-* mutant virus, even after extended exposure (Fig 10A lane 2a). However, dsDNA levels increased until they were ~1.5 fold greater than wild type by 72 hpi, although little or no viral ssDNA was observed (Fig 10A lanes 4 and 6). This is reminiscent of reduced ssDNA accumulation following transient replication of TGMV *cp-* and *al2-* mutants in protoplasts [47], and suggests diminished CP levels.

For CaLCuV, a similar delayed accumulation of viral DNA was observed for the *cle-* mutant virus compared to wild type. However, both dsDNA and ssDNA levels increased until they were ~1.6 fold greater than wild type after 72 hpi (Fig 10B). Why a loss of ssDNA was observed upon CLE mutation in TGMV but not CaLCuV is not clear. However, delayed accumulation of viral DNA for both mutant viruses is consistent with the observed delays in symptom appearance (Table 1) (Table 2). In any case, the results suggest that the differences observed in CLE mutant compared to wild type DNA-A are most likely due to altered gene expression.

### CLE mutation correlates with reduced accumulation of CP mRNA

To determine if CLE mutation impacts *CP* promoter activity, total RNA was isolated 48- and 72 hpi from the same *N*. *benthamiana* tissue used to analyze TGMV DNA levels (Fig 10A). Isolated RNA was used to generate cDNA, qPCR was performed using primers specific for the TGMV CP coding sequence, and CP mRNA copy number was calculated using a standard curve (S4 Table). After adjusting for the amount of potential dsDNA template, CP mRNA levels for TGMV *cle*- were two-fold less than TGMV wild type at 48 hpi, and six-fold less at 72 hpi (Fig 10C). The reduced amount of CP mRNA, and presumably CP, is consistent with the delay and attenuation of symptoms in plants infected with the TGMV *cle-* mutant virus. A similar delay and attenuation were previously observed when mutations were introduced into the *CP* coding sequence [48].

CP mRNA levels were compared following whole plant infections with CaLCuV wild type and CaLCuV *cle*-, both of which were delivered to plants by agroinoculation along with DNA-B. Transcript levels were normalized to PP2A, and further adjusted for viral DNA levels determined by qPCR. In symptomatic tissue infected with the *cle-* mutant, CP mRNA levels were reduced compared to wild type to a level that approached statistical significance (Fig 8E). Reduction was far greater in asymptomatic tissue from plants infected with CaLCuV *cle-*, in part because it is likely that a larger proportion of viral genomes in this tissue are transcriptionally silenced by host defense. Thus, in both viruses and both hosts, loss of the TCP24 binding site (CLE) had the effect of reducing CP mRNA levels, most likely reflecting a reduction in *CP* promoter activity.

### Mutation of the CLE results in decreased deposition of H3K27me3

We previously detected H3K27 methylation, a hallmark of facultative gene repression and a read-out for PRC2 activity, on CaLCuV chromatin from infected *Arabidopsis* plants [4,6]. Because PRC2 is often recruited by repressive transcription factors [49,50], we asked whether TCP24 might have some role in recruiting PRC2. Unfortunately, a TCP24 mutant is not yet available, and in any case TCP family proteins could exhibit some functional redundancy. Consequently, H3K27me3 levels on wild type viral chromatin were compared with levels on chromatin of *cle-* mutant viruses lacking a TCP24 binding site. To do this, ChIP was performed using H3K27me3 antibody and nuclear extracts obtained from plants agroinoculated with wild type or *cle-* TGMV or CaLCuV DNA-A, along with cognate wild type DNA-B. Extracts were sonicated under conditions that shear DNA into ~200 bp fragments. The regions examined spanned the viral IR with four (TGMV) or five (CaLCuV) ~100 bp amplicons, using PCR primers specific for the DNA-A components of TGMV or CaLCuV. This was assured by having at least one primer from each set located outside the common region.

**Fig 11:**
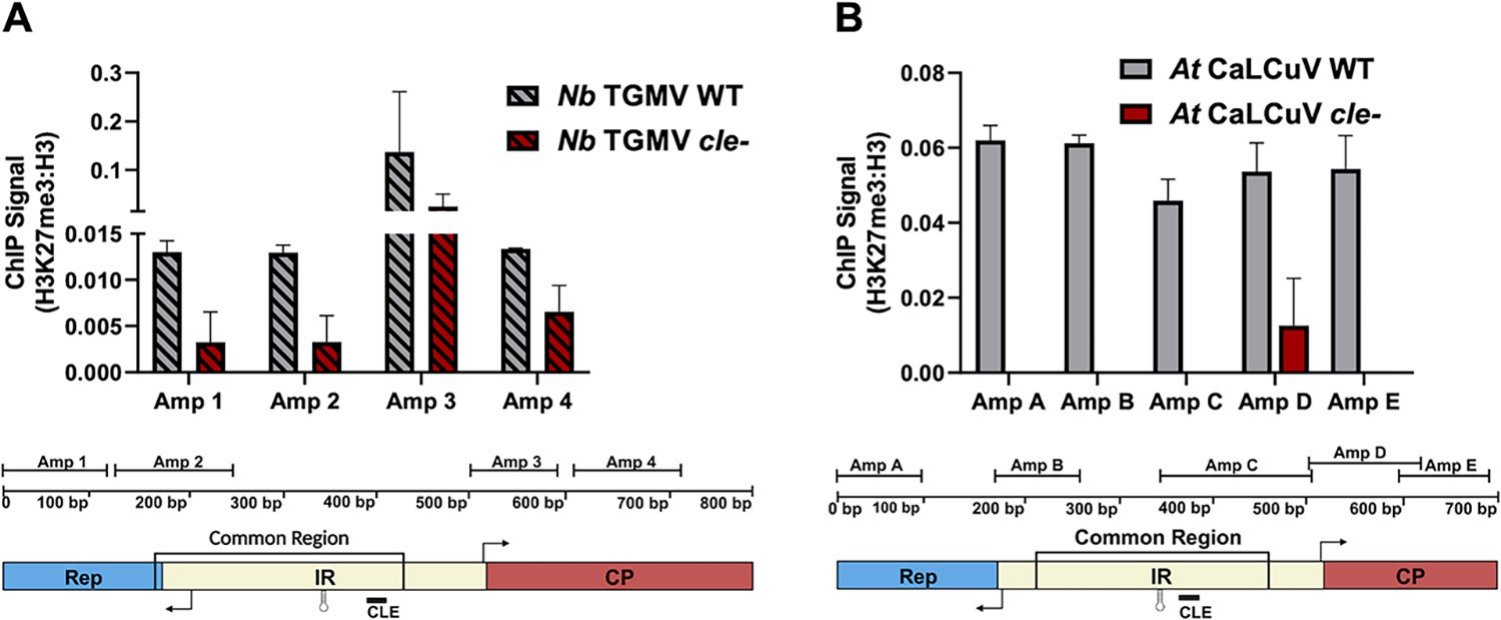
CLE mutation decreases H3K27me3 levels on the viral IR. ChIP-qPCR experiments were performed with H3K27me3 antibody using nuclear extracts from *N*. *benthamiana* or *Arabidopsis* plants systemically infected with wild type (WT) or *cle-* TGMV or CaLCuV. Tissue from symptomatic plants was pooled for analysis. Data were normalized to input DNA, with signal from the negative control IgG immunoprecipitate subtracted. Values were also normalized to ChIP-qPCRs performed with histone H3 antibody using the same extracts. Amplicon regions (~100 bp) are depicted on diagrams that show the positions of the intergenic region (IR), conserved hairpin, the common region, and the Rep and CP ORFs with transcription start sites (right angle arrows). (A) ChIP with extracts from *N*. *benthamiana* (*Nb*) plants infected with wild type TGMV or TGMV *cle-*. Three replicates are shown, with standard error of the mean. (B) ChIP with extracts from *Arabidopsis* (*At*) infected with wild type CaLCuV or CaLCuV *cle-*. Two replicates are shown, with standard error of the mean.

ChIP-qPCR with extracts from *N*. *benthamiana* plants infected with wild type TGMV revealed enrichment of H3K27me3 (PRC2 footprint) across the IR and extending into flanking coding regions (Fig 11A). However, when ChIP was carried out with extracts from plants infected with the TGMV *cle-* mutant, H3K27me3 association was substantially reduced. Similar results were obtained in experiments with wild type and *cle*- CaLCuV in *Arabidopsis* plants. Again, H3K27me3 signal was evident on all IR amplicons from wild type CaLCuV, while little or no signal was observed on CaLCuV *cle-* chromatin (Fig 11B).

ChIP was also performed with wild type and *cle*- CaLCuV in *N*. *benthamiana* plants. As before, DNA-A components were co-inoculated with wild type DNA-B. These experiments employed the same CaLCuV DNA-A amplicons (A-E) used to analyze H3K27me3 levels in *Arabidopsis*, and also included an amplicon specific for the CLE region of DNA-B (B-IR Right). Similar to results with TGMV in this host, H3K27me3 signal was evident on all DNA-A IR amplicons from wild type CaLCuV, while levels were substantially reduced on *cle-* chromatin. In addition, ChIP-qPCR with the B-IR Right amplicon demonstrated substantial H3K27me3 deposition on wild type DNA-B chromatin regardless of whether infections were generated by wild type or *cle-* DNA-A (S9 Fig). Thus, these experiments revealed similar reductions in H3K27me3 levels on CaLCuV and TGMV *cle-* DNA-A chromatin in the same host. As DNA-B also contains a CLE sequence, the results further suggest that the *CP* and *NSP* promoters are similarly regulated, although this question requires further study.

*Arabidopsis* has three H3K27 methyltransferases in different PRC2 complexes: CURLY LEAF (CLF), SWINGER (SWN), and MEDEA (MEA). CLF and SWN are partially redundant in most tissues, whereas MEA functions in the female gametophyte and developing seeds [24,25]. CLF is considered the major H3K27me3 writer in most tissues, and an *Arabidopsis* mutant lacking CLF (*clf-28*) exhibits reduced levels of H3K27me3 on cellular chromatin, although consistent with a redundant role for SWN, this modification is not eliminated [51,52]. While *clf-28* plants have a developmental phenotype and are somewhat stunted, following inoculation with wild type CaLCuV it was nevertheless possible to observe that disease symptoms were significantly attenuated on *clf-28* plants compared to wild type plants, although viral DNA and CP mRNA levels were not significantly different (S10 Fig). Changes in host susceptibility do not always correlate with differences in geminivirus DNA levels, as we have previously observed [4,6]. Experiments employing mutant viruses versus mutant plant hosts can yield different outcomes in this respect, especially if functional redundancy is present, as is the case here. Nevertheless, symptom attenuation in *clf-28* plants is consistent with symptom attenuation observed with *cle-* viruses, and further supports a role for PRC2 in viral gene regulation.

Together, the results of ChIP experiments with wild type and *cle-* viruses demonstrate that H3K27me3 writer PRC2 interacts with TGMV and CaLCuV chromatin, and that mutation of the CLE, the TCP24 binding site, greatly reduces H3K27me3 levels.

## Discussion

Using a genetic approach with the TGMV *CP* promoter sequence as a target, we identified a plant specific DNA-binding protein, AtTCP24, from an *Arabidopsis* cDNA library. AtTCP24 is a member of the repressive class II TCP family. Using EMSA and ChIP we showed that TCP24 protein is able to specifically bind proximal TGMV and CaLCuV *CP* promoter sequences both *in vitro* and *in vivo*. However, TCP24 does not interact with sequences that act to repress the *CP* promoter in phloem tissue. Thus, TCP24 binding is specific for sequences located between -125 bp and -107 bp upstream of the *CP* TSS, which includes the conserved late element (CLE). The binding sites of class II TCP proteins have been characterized and match the CLE found in the late promoters of many begomoviruses (GTGGNCCC vs. GTGGTCCC) [34,53]. Mutation of the CLE abolished TCP24 binding to TGMV and CaLCuV *CP* promoter sequences in EMSA assays, providing compelling evidence that binding is mediated by this sequence. Importantly, we also found, by protein-protein gel blot, BiFC analysis, and Co-IP, that TCP24 interacts with viral AL2 protein in the nucleus. AL2 is required for *CP* promoter activity but does not by itself bind DNA, and a goal of this study was to identify host factors capable of interacting with this essential viral transactivator. TCP24-AL2 interaction strongly suggests that TCP24 can target AL2 to responsive promoters.

CLE mutation also severely reduced the ability of TGMV and CaLCuV to establish systemic infection in *N*. *benthamiana* and *Arabidopsis*. Infections were often extremely mild or asymptomatic, mean latent periods were increased, and significantly less viral DNA accumulated in systemic tissues infected with TGMV *cle-* or CaLCuV *cle*- compared to cognate wild type viruses. However, in transient replication assays carried out in *N*. *benthamiana* leaves and involving only DNA-A, replication of *cle-* mutant viruses was delayed but attained levels similar to or greater than wild type by 72 hpi. Thus, mutation of the CLE sequence in both TGMV and CaLCuV did not block viral DNA replication, and so the reduced infectivity observed in *N*. *benthamiana* and *Arabidopsis* is most likely attributable to disruption of *CP* expression leading to reduced systemic spread. The case for dysregulation is supported by results showing that CP mRNA levels were reduced up to 6-fold for TGMV *cle-* mutants relative to wild type virus. Further, mutation of the TGMV CLE resulted in a nearly complete loss of ssDNA, consistent with reduced levels of CP which could sequester ssDNA. For reasons currently unknown, ssDNA levels were not reduced in transient assays with CaLCuV *cle*-. However, CP mRNA levels were reduced in *Arabidopsis* plants infected with CaLCuV *cle*- DNA-A and wild type DNA-B, despite the presence of DNA-B encoded NSP which can also bind ssDNA [54].

An analysis of the *CP* and *NSP*/*BR1* promoters of >40 begomo- and curtoviruses for the presence of class II TCP binding sites revealed some interesting observations. While 18 viruses have at least one TCP binding site in the *CP* promoter, and 15 have one in the *NSP* promoter, only 13 of the 20 bipartite viruses examined have a TCP binding site in both promoters. Also, while the TCP motif can be found on either the viral or complementary sense DNA strand, they are always on the same sense strand when present in both the *CP* and *NSP* promoters. This could imply different mechanisms for regulation of the two promoters in different viruses, with a common control mechanism for *CP* and *NSP* when a TCP binding site is present in both promoters and different mechanisms if a binding site is found in only one promoter.

To date we have identified two AL2-interacting host transcription factors, PPD2 and TCP24, that also interact with the intergenic region of two bipartite geminiviruses. The interplay between these factors remains to be elucidated, but both could be involved in recruiting AL2, which might explain why *CP* expression is not completely lost in TGMV and CaLCuV *cle*- mutants. Further, both PPD2 and TCP24 are repressive factors that regulate leaf development and aspects of the cell cycle to promote differentiation [37,55–58]. Geminiviruses require host DNA synthesis machinery for viral DNA replication, and its absence in differentiated cells that have exited the cell cycle may constitute a barrier to infection [59]. Thus, we speculate that geminiviruses have evolved mechanisms to oppose the activity of these repressive transcription factors through the action of AL2 protein (see below).

Insights into how TCP24 functions are provided by studies that demonstrate interactions with several different cellular proteins. For example, in concert with AtABAP1, TCP24 acts to repress the expression of pre-replication complex genes, including AtCDT1 [37]. TCP24 has also been shown to interact with TCP INTERACTOR CONTAINING EAR MOTIF PROTEIN1 (TIE1), which can recruit the TOPLESS (TPL) co-repressor [57]. EAR motif-containing transcription factors (e.g., FLC, AS1) can directly recruit TPL or TOPLESS related factors (TPR), which provide a bridge between transcription factors, histone deacetylase, and chromatin remodeling complexes to create a repressive chromatin environment [60–62]. Repressive transcription factors that lack an EAR domain (like TCP24) bind EAR motif-containing adaptor proteins (e.g., TIE1 and NINJA) to indirectly recruit TPL/TPR [63]. That TCP24 binds TIE1 supports its role as a repressive transcription factor.

Recently, several TPL-associated transcription factors have been linked to PRC2 by interaction with LIKE HETROCHROMATIN PROTEIN1 (LHP1), which binds H3K27me3 [50,58]. This in turn prompted us to examine H3K27me3 levels during infection with wild type and *cle*- TGMV and CaLCuV. ChIP experiments with extracts from infected plants demonstrated that H3K27me3 is enriched across the IR in wild type virus chromatin. Remarkably, ChIP further revealed that mutation of the CLE significantly reduced H3K27me3 deposition on DNA-A. Coupled with highly attenuated infections by *cle*- mutant viruses, these observations indicate that PRC2-enforced repression is an important aspect of the viral gene expression program. In this context, it is important to note that if PRC2-mediated repression were a component of antiviral defense, reducing H3K27me3 levels on viral chromatin would be expected to render plants hypersensitive to infection.

In summary, we present evidence that TCP24 interaction with the CLE leads to the establishment of a repressive chromatin landscape including nucleosomes modified with H3K27me3, although it is not presently known if PRC2 is directly or indirectly recruited by TCP24. We further show that TCP24 interacts with AL2, and that CLE mutation results in reduced CP mRNA levels. This suggests the interaction targets AL2, which is required for derepression and activation of the *CP* promoter. Thus, in *cle*- mutant viruses, loss of a repressive chromatin mark (H3K27me3) counter-intuitively coincides with a reduction in CP mRNA levels. Based on these findings, we hypothesize that a repressive host transcription factor (TCP24) is repurposed to target a viral activator (AL2) needed to drive *CP* expression. Whether TCP24-AL2 interaction occurs on viral chromatin and whether this interaction supports the establishment of transcriptionally permissive chromatin is currently under investigation. In any case, it seems likely that opposing viral chromatin landscapes exist at different times during the infection cycle, and possibly on different viral chromosomes. While our hypothesis has the virtue of simplicity, reality is probably more complex. In this regard, it was recently shown that abscisic acid (ABA) response elements in mungbean yellow mosaic virus (MYMV), another bipartite begomovirus, are required in addition to the CLE for activation of the *NSP*/*BR1* promoter [64]. While ABA response elements are not present in the *CP* promoters of TGMV, CaLCuV, or MYMV, it is likely that as yet undiscovered activators and co-activators are involved in *CP* expression.

Finally, because all class II TCPs bind the same sequence, we cannot rule out the possibility that at least some function redundantly with TCP24 during geminivirus infection. With this in mind, it is interesting to speculate that geminiviruses have evolved a strategy, via AL2, to derepress and activate both viral and cellular genes repressed by class II TCPs for the dual purpose of regulating viral gene expression and creating an environment conducive to virus replication.

## Methods

### Construction of reporter yeast strains and yeast one-hybrid screening

Generation of yeast strains containing four tandemly repeated copies of sequences required for activation of the *CP* promoter (YM4271-TGMVCPactivator) has been previously described [22,65]. For one-hybrid screening, YM4271-TGMVCPactivator was transformed with pAD-GAL4/cDNA plasmids from an *Arabidopsis* cDNA library according to the Matchmaker One-Hybrid System protocol (Clontech Laboratories, Mountain View, CA). Transformants were identified and plasmid DNA containing *Arabidopsis* cDNA sequences purified and sequenced as described [22]. cDNA sequences were identified by BLAST analysis using The *Arabidopsis* Information Resource (TAIR) database.

### Construction of target entry clones

All primer sequences are given in S5 Table. Primers specific for AtTCP24 (TCP24entrF + TCP24entrR) or AtERF13 (AtERF13entrF + AtERF13entrR) were used to amplify coding regions by RT-PCR using total RNA isolated from *Arabidopsis* as template. A second AtTCP24 PCR product containing an 18 bp linker sequence coding for six alanine residues was generated using primers AtTCP24entrLinkerF + AtTCP24SnabR. Primers specific for eGFP (eGFPentrF + eGFPR) were used to amplify the coding sequence by PCR. Amplified products of 975 bp (TCP24), 996 bp (TCP24Linker), 698 bp (ERF13) and 720 bp (eGFP) were gel-purified and introduced into the pENTR/SD/D-TOPO gateway vector (Invitrogen, Carlsbad, CA) according to the manufacturer’s instructions. Recombinant entry clones AtTCP24Entr, AtTCP24EntrLinker, AtERF13Entr, and eGFPEntr, were verified by sequencing. An entry clone containing TGMV AL2 has been previously described [44].

### Gateway cloning into destination vectors

Entry clones were introduced into the appropriate Gateway compatible LR recombination reactions using ClonaseII according to the manufacturer’s instructions (Invitrogen). Entry clones containing AtTCP24 and TGMV AL2 were introduced into the Gateway compatible pEARLYGATE 104 binary vector to generate N-terminal translational fusions to YFP for subcellular localization (YFP-AtTCP24 and YFP-TGMVAL2). Entry clones containing AtTCP24, and AtTCP24Linker were introduced into BiFC binary vectors pSITE-nEYFP-C1 or pSITE-cEYFP-C1 [45]. An ENTRY clone containing TGMV AL2 was introduced the Gateway compatible pEARLYGATE 202 binary vector to generate an N-terminal translational fusion to FLAG (pEG202-AL2) for subsequent cloning into pTRBO. Destination clones were subsequently transformed into *A*. *tumefaciens* LBA4404 by electroporation as described [66].

### Cloning of tandemly repeated copies of the DNA-A genome component of TGMV and CaLCuV

The 1462 bp EcoRI-ScaI fragment from pTGA26 was cloned into the EcoRI-ScaI sites of the plant binary vector pLSU-1 [67] (kindly provided by Dr. Wayne Curtis, Penn State University). The resulting clone (TGMV A 0.56mer) lacks the common region from TGMV DNA-A. The genome-length 2588 bp EcoRI fragment from pTGA26 was then cloned into the EcoRI site of TGMV A 0.56mer to generate pGS2052, which contains tandemly repeated 1.56 copies of the wild type TGMV DNA-A genome component, with a single common region and a single copy of the conserved late element (CLE). To generate a mutation in the TGMV CLE sequence, a 2026 bp fragment was amplified by PCR with primers TGMVcleFBgl + TGMV SacIR using pGS2052 as template. The resulting fragment was restricted with KpnI and SacI and cloned into pLSU-1 restricted with KpnI and SacI to generate TGMV A 0.78*cle-*. The construct contains a mutation of the CLE sequence by introducing a BglII restriction site. A second 2061 bp fragment was amplified by PCR with primers TGMVcleRBgl + TGMV KpnF using pGS2052 as template. The resulting fragment was restricted with BglII and KpnI and cloned into TGMV A 0.78*cle-* restricted with BglII and KpnI to generate pGS2056. The resulting construct has tandemly repeated 1.6 copies of the TGMV DNA-A genome component, containing a single common region, with a mutation in the TGMV CLE sequence.

The 1219 bp EcoRI-SacI fragment of CaLCuV DNA-A in pUC19 [10] was cloned into the EcoRI-SacI sites of the plant binary vector pLSU-1. The resulting clone (CaLCuV A 0.47mer) lacks the common region from CaLCuV DNA-A. The genome-length 2583 bp SacI fragment of CaLCuV DNA-A was then cloned into the SacI site of CaLCuV A 0.47mer to generate pGS2062, which contains tandemly repeated 1.47 copies of the wild type CaLCuV DNA-A genome component, with a single common region and a single copy of the CLE. To generate a mutation in the CaLCuV CLE sequence, a 2451 bp fragment was amplified by PCR with primers CabcleXhoF + CabSacIR using pGS2062 as template. The resulting fragment was restricted with XhoI and SacI and cloned into pLSU-1 restricted with XhoI and SacI to generate CaLCuV A 0.95*cle-*. A mutation of the CLE sequence was constructed by introducing an XhoI restriction site. A second 1363 bp fragment was amplified by PCR with primers CabcleXhoR + CabDIIIF using pGS2062 as template. The resulting fragment was restricted with XhoI and HindIII and cloned into CaLCuV A 0.95*cle-* restricted with XhoI and HindIII to generate pGS2055. The resulting construct has tandemly repeated 1.48 copies of the CaLCuV DNA-A genome component, containing a single common region, with a mutation in the CaLCuV CLE sequence. Constructs containing the wild type and *cle-* mutant viruses were transformed into *Agrobacterium* LBA4404 by electroporation as described [66].

### Protein expression and purification

For protein expression in *E*. *coli*, the coding region for AtTCP24 was amplified by PCR with primers AtTCP24BamF and AtTCP24SnabR using AtTCP24Entr as template. The PCR product was restricted with BamHI and SnaBI and cloned into pGEX2T restricted with BamHI and SmaI. The resulting cloned DNA is capable of expressing an N-terminal GST-tagged protein. Cloned DNA (pGST-AtTCP24) was expressed in *E*. *coli* strain Rosetta (Novagen-MilliporeSigma, Burlington, MA), protein fractions bound to glutathione-agarose, and purified GST or GST-TCP24 eluted in 250 μl fractions as described [11]. Equivalent amounts of GST and GST-TCP24 in the eluted fractions was determined by Western blot analysis using an anti-GST antibody (Santa Cruz Biotechnology, Dallas, TX). The TGMV AL2 coding region was amplified by PCR (AL2FBgl + AL2INTEIN3’) using pTGA26 as template and cloned into the BamHI and EcoRI sites of pET28a(+) (ThermoFisher Scientific, Waltham, MA) to produce pET28a(+)-AL2. The resulting cloned DNA can express an N-terminal 6xHis-tagged AL2 protein. The AtTCP24 coding region was amplified by PCR (AtTCP24BamNcoF + AtTCP24XhoR) using pGAD-AtTCP24FL as template and cloned into the BamHI and XhoI sites of pET28a(+) to produce pET28a(+)-AtTCP24. The resulting cloned DNA can express an N-terminal 6xHis-tagged TCP24 protein. The eGFP coding region was introduced into the pET300/NT-DEST vector (Invitrogen) using the eGFPEntr clone. The resulting cloned DNA can express an N-terminal 6xHis-tagged eGFP protein. 6xHis-tagged proteins were expressed in *E*. *coli* Rosetta cells and purified using Ni-NTA agarose (Invitrogen) essentially as described [68]. Pellets from bacterial cultures were resuspended in solubilization solution (8M urea and 100 mM Tris-HCl, pH 8) and incubated for 30 min at room temperature with shaking. After centrifugation, 0.5 ml of Ni-NTA agarose beads was added to the clarified cell lysate (soluble fraction). After incubation for 1 h at room temperature, mixtures were loaded to Poly-Prep Chromatography Columns (Bio-Rad, Hercules, CA), and beads washed with solubilization solution to remove unbound proteins. Beads were washed with 1:1 and 1:3 dilutions of solubilization solution and resuspended in renaturation solution (20 mM Tris-HCl, pH 8, 20 mM NaCl and 20 mM imidazole), and incubated for 1 h on ice with gentle shaking. His-tagged proteins were eluted using 1 ml of elution buffer (20 mM Tris-HCl, pH 8, 20 mM NaCl and 200 mM imidazole). The presence of 6xHis-tagged proteins was confirmed by Western blot analysis using anti-6xHis antibody. GST and GST-TCP24 were expressed in *E*. *coli* Rosetta cells and purified using Pierce Glutathione Superflow Agarose (ThermoFisher Scientific) essentially as described previously [11], with some modifications. Cell lysis was performed using B-PER Bacterial Protein Extraction reagent (ThermoFisher Scientific) and following centrifugation, the cell lysate containing a soluble fraction of GST protein was stored and used for Far Western analysis. The majority of the GST-TCP24 protein was present in the insoluble fraction (pellet) after lysis in B-PER reagent (ThermoFisher Scientific). The pellet was resuspended in lysis buffer (50 mM Tris-HCl, pH 7, 250 mM NaCl, 1% Triton X-100, 1 mM EDTA, 10% glycerol, 1 mM DTT, 1 mM PMSF, 2 M Urea) and incubated on ice for 30 min. Following centrifugation, the pellet was resuspended in solubilization solution as described above for 6xHis-tagged protein purification and incubated for 30 min at room temperature. The lysate was centrifuged, and the soluble supernatant fraction dialyzed overnight (Slide-A-Lyzer MINI Dialysis unit, 3.5K MWCO (ThermoFisher Scientific) at 8°C in 50 mM Tris-HCl, pH 7, 250 mM NaCl, 10% glycerol, 1 mM DTT. The dialyzed sample containing soluble GST-TCP24 protein was used for Far Western analysis. The presence of GST and GST-TCP24 was confirmed by Western blot analysis using anti-GST antibody.

For protein expression in *N*. *benthamiana* plants, the TMV-based transient expression vector pTRBO was utilized [42]. The GST-tagged coding region for AtTCP24 was amplified by PCR with primers GSTPacIF and AtTCP24NotIR using pGST-AtTCP24 as template. The GST-tagged coding region for AtERF13 was amplified by PCR with primers GSTPacIF and AtERF13 NotIR using pGST-AtERF13 as template. The 674 bp fragment containing the GST coding region was amplified by PCR with primers GSTPacIF and GSTSnaNotR using GEX-2T as template. The 1068 bp fragment containing GST-AL2 was amplified by PCR with primers GSTPacIF and TGMVAL2NotIR using pGEX-TrAP [11] as template. The 492 bp fragment containing FLAG-AL2 was amplified by PCR with primers 3xFLAGPacF and AL2NotIR using pEG202-AL2 as template. Amplified DNA fragments were restricted with PacI and NotI and cloned into pTRBO restricted with PacI and NotI. The resulting constructs (pTRBO-GSTAtTP24 and pTRBO-GSTAtERF13) were transformed into *Agrobacterium* LBA4404 by electroporation as described [66].

### Electrophoretic mobility shift assay (EMSA)

All DNA fragments were generated by PCR, and the resulting products gel purified. Binding of 6xHis- or GST-tagged AtTCP24 protein to CP regulatory sequences was examined by EMSA. Unlabeled DNA fragments containing the TGMV IR with wild type (WT) or mutated (*cle-*) CLE sequences were amplified by PCR (TGMVAL1NcoFnew + TGMVCREcoR) using pLSU1 TGMV A 1.5 (one IR) and pLSU1 TGMV A 1.5*cle*^*-*^ as template, respectively. Unlabeled DNA fragments containing the CaLCuV IR with a wild type (WT) or mutated (*cle-*) sequence were amplified by PCR (CbLCVCCRKpnF and CbLCVCCREcoR) using pGS2101 and pGS2102 as template, respectively. Unlabeled DNA fragments from TGMV (105 bp) and CaLCuV (144 bp) containing sequences that repress the *CP* promoter in phloem tissue (distal phloem repressor element) [8,10] were amplified using TGMVRP7v + TGMVRP5c and CLCVRep5 + CLCVRep3, respectively. TGMV or CaLCuV DNA fragments containing the TGMV or CaLCuV IR with a wild type (WT) CLE sequence labeled with FITC were amplified by PCR with a primer labeled at the 5’-end with FITC (TGMVAL1NcoFnew + TGMVIRrfitc or CbLCVCCRKpnF + CaLCuVIRAcpFITC) using pLSU1 TGMV A 1.5 (one IR) or pGS2101 as template. The multiple cloning site of the pENTR/SD/D-TOPO gateway vector was amplified by PCR (M13F and M13R) for use as a non-specific competitor DNA. PCR products were purified using the QIAquick PCR purification kit (Qiagen) according to the manufacturer’s instructions. Probe DNAs were incubated with 250 ng poly(dI:dC) and 0–1.25 μg of competitor DNA in binding buffer (50 mM Tris–HCl, pH 7.2, 5 mM MgCl_2_, 2.5 mM EDTA, 2.5 mM DTT, 250 mM NaCl, 20% glycerol) in the presence of equivalent volumes of eluted GST or GST-TCP24 for non-radiolabeled probe DNAs (25 ng), or 0.5–1.0 μg 6xHis-TCP24 for FITC-labeled probe DNAs (25 ng). Reactions were incubated at room temperature for 25 min and complexes resolved by electrophoresis in 2% agarose gels in 0.5X TB buffer or 6% polyacrylamide TBE Gels (Invitrogen). After electrophoresis, gel shifts were analyzed using the iBright 1500 Imaging System (Invitrogen) for FITC-labeled probes (acrylamide gels) or by ethidium bromide staining (agarose gels) for unlabeled probe DNAs.

### Far Western analysis

Far Western was performed for protein-protein interaction analysis as described [43]. Three identical samples of eluted fractions of 6xHis-TCP24, 6xHis-AL2, and 6xHis-GFP were resolved on 4–20% Mini-PROTEAN TGX Precast Protein Gels (Bio-Rad) and transferred to nitrocellulose membrane. Equivalent protein amounts were calculated based on ELISA using anti-6xHis antibody. The three identical blots containing immobilized 6xHis-TCP24, 6xHis-AL2, and 6xHis-GFP were incubated with blocking buffer (100 mM Tris-HCl, pH 8, 0.25% BSA, 0.25% gelatin, and 0.3% Tween-20), and one blot probed with anti-6xHis antibody (1:75000 dilution) to verify the presence of 6xHis-TCP24, 6xHis-AL2 and 6xHis-GFP. The other two blots were incubated with 15 μg soluble protein extracts from *E*. *coli* cells expressing either GST or GST-TCP24 overnight at 4°C. Both membranes were probed with anti-GST antibody (1:75000 dilution). Images were taken using the iBright 1500 Imaging System (Invitrogen).

### Microscopic analysis of GFP fluorescence

BiFC and localization analyses were assessed by fluorescence microscopy. For microscopic observations, discs from infiltrated leaves were mounted on microscope slides and analyzed for YFP, RFP, or eGFP expression using a fluorescent microscope (Axioskop, Carl Zeiss, Oberkochen Germany; or EVOS5000 Imaging System, Invitrogen) as described [44]. Samples were imaged with a 40X objective, using YFP (excitation 485 nm—emission 515 nm) and RFP (excitation 545 nm—emission 620 nm) filter sets. Images are representative of 3 independent experiments where five discs (~ 5mm) were taken from each infiltration site, and three fields observed per disc, for a total of 15 fields per sample. Each field has 96 cells, with an area of 50 x 50 μm^2^ per cell.

### Co-Immunoprecipitation (Co-IP)

*N*. *benthamiana* leaves were co-infiltrated with *Agrobacterium* containing pTRBO-FLAG::AL2, and either pTRBO, pTRBO-GST, pTRBO-GST::AL2, or pTRBO-GST::TCP24. Following incubation for 48 hr, the infused area from each leaf (total of 12 leaves) was harvested and fixed with formaldehyde, and chromatin was purified using the ChIP Kit for Plants (Abcam, Cambridge, UK). Following purification, chromatin was resuspended in Co-IP buffer (50 mM Tris-HCl, pH7.5, 150 mM NaCl, 5 mM EDTA, 0.1% Triton X-100, 10% glycerol) and sonicated for 30 min using a 10 sec on /10 sec off cycle at 50% power for 30 min using a Qsonica Sonicator (Qsonica, Newtown, CT). Following centrifugation at 12,000 x g for 10 min at 4° C, soluble chromatin was used for co-immunoprecipitation reactions. Samples were diluted 40x using 16.7 mM Tris-HCl, pH8.0, 167 mM NaCl, 1.2 mM EDTA, 1.1% Triton X-100, 10 μg/ml BSA, 1x Sigma Plant Protease Inhibitor, and pre-cleared using Pierce protein A/G agarose plus (ThermoFisher Scientific, Rockford, IL) for 2 hr at 4° C. Pre-cleared samples were added to 50 μl Anti-M2 FLAG Affinity Agarose Beads (Millipore Sigma, St. Louis, MO) and incubated on a rotator at 4°C overnight. Beads were washed five times with 20 bed volumes of Co-IP buffer followed by five times with 20 bed volumes of Co-IP buffer containing 500 mM NaCl. Immunoprecipitated proteins were eluted using 100 μl 0.1 M glycine, pH 3.5, at room temperature for 10 min, neutralized by adding to 10 μl 0.5 M Tris, pH8.0, 1.5 M NaCl, and analyzed by Western Blot. Blots were probed with either rabbit anti-GST (Genscript, Piscataway, NJ) or rabbit anti-FLAG (Millipore Sigma) primary antibodies and WesternSure goat anti-rabbit HRP secondary antibody (LicorBio, Lincoln, NE). Following development with Pierce ECL Western Blotting substrate, chemiluminescent signals were detected using the Invitrogen iBright CL1500 Imaging System (ThermoFisher Scientific).

### Chromatin immunoprecipitation (ChIP)

Chromatin was purified from leaves of transgenic *N*. *benthamiana* plants containing a TGMV *CP* promoter-GUS reporter (pTGA55M) 48 hr after infiltration with *Agrobacterium* cultures capable of expressing GST-TCP24 or GST-ERF13 as described [10]. Purified chromatin was sonicated five times for 20 sec each at 40% duty cycle and 20% power, using an EpiShear Probe Sonicator (Active Motif, Carlsbad, CA). To determine the quality of sonication, end-point PCR was performed using primers specific for either the activation element of the TGMV *CP* promoter alone (TGMVCPReg5 + GUSseq) or for the *CP* promoter fused to the GUS reporter gene (TGMVCPReg5+ GUSR). Soluble chromatin was used for immunoprecipitation reactions in two independent experiments using anti-GST, anti-RFP, or anti-GUS antibodies (Santa Cruz Biotechnology) overnight at 4° C. After capture using Protein A/G agarose, non-bound material was removed by successive washes and immunocomplexes eluted twice from the beads using elution buffer (1% SDS, 0.1 M NaHCO_3_). The cross-links were reversed by incubation overnight at 65°C in the presence of 5 M NaCl, followed by treatment with proteinase K to remove protein. DNA was purified by phenol-chloroform extraction and recovered by ethanol precipitation. Precipitated DNA was resuspended in 10 mM Tris-HCl, pH 8.0, 1 mM EDTA and differences in the amount of DNA isolated assessed by qPCR with primers (TGMVCPReg5 + TGMVCPReg3) specific for the proximal TGMV *CP* promoter (Fig 1) using a 7500 Real-time PCR system (Applied Biosystems, Foster City, CA) with SYBR green. DNA was also purified from soluble chromatin prior to immunoprecipitation to provide input chromatin samples.

For H3K27me3 analysis, ChIP was performed with tissue isolated from systemically infected plants essentially as described [69]. Chromatin was purified from inflorescences harvested at 18 dpi from *Arabidopsis* infected with CaLCuV or CaLCuV *cle*-, or from leaves harvested at 14 dpi from *N*. *benthamiana* infected with TGMV or TGMV *cle*-. Isolated nuclei were sonicated using the Covaris E220 system (Covaris, Woburn, MA). Soluble chromatin was immunoprecipitated overnight with anti-IgG (Millipore 12–371, Burlington, MA), anti-H3 (GenScript A01502, Piscataway, NJ) or anti-H3K27me3 antibodies (Millipore 07–449 or Abcam ab6002, Waltham and Boston, MA). Immunocaptured viral DNA was quantitated by qPCR using amplicon primers. Data were normalized to input DNA, with signal from the negative control IgG immunoprecipitate subtracted. Values were also normalized to ChIP-qPCR performed with histone H3 antibody using the same extracts.

### Plant inoculation and analysis

Procedures for inoculating *N*. *benthamiana* or *Arabidopsis* plants with *Agrobacterium* cultures containing tandemly repeated copies of TGMV DNA-A and DNA-B, and CaLCuV DNA-A and DNA-B, using a standard dose (OD_600_ = 1.0) have been described previously [10,70]. Inoculated *N*. *benthamiana* plants were scored for appearance of symptoms typical of a TGMV or CaLCuV infection and mean latent periods estimated as described [70]. For *Arabidopsis* plants, the severity of infection was scored using the following infectivity rating scale: 0 = no symptoms; 1 = mild silique/floral deformation on a single bolt, no stunting; 2 = mild to moderate silique/floral deformations on multiple bolts, no stunting; 3 = moderate silique/floral deformations on multiple bolts, mild to moderate stunting; 4 = major deformations of silique and flowers, severe stunting or no growth. Significance of differences observed in mean latent period and average symptom scores was confirmed by a two-tailed Student’s *t*-test. DNA was isolated from *N*. *benthamiana* or *Arabidopsis* leaves away from the site of inoculation in individual plants as described [71]. The presence of replicating viral DNA in *N*. *benthamiana* plants was assessed by quantitative real-time PCR using primers specific for TGMV (TGMVqPCRNbF+ TGMVqPCRNbR) or CaLCuV (CaBqPCRNbF+ CaBqPCRNbR), and analysis performed using a 7500 Real-time PCR system (Applied Biosystems) with SYBR green. For each experiment, the absolute amount of viral DNA was determined using a standard curve generated from a PCR product specific for the CP ORF of TGMV or CaLCuV. Five to seven independent biological samples from individual plants were used for the analysis and for each biological sample three technical replicates were included. To assess differences in viral DNA loads in different viral treatments, an Analysis of Variance (ANOVA) was performed followed by a Tukey-Kramer post-hoc comparison. For *Arabidopsis*, the presence of viral DNA was detected by qPCR using primers specific for CaLCuV (CaBqPCRAtF+CaBqPCRAtR), with analysis performed using the StepOnePlus system (Applied Biosystems) with SYBR Green. To assess differences in viral DNA levels, a relative analysis was performed using the ΔΔCt method and normalizing levels to 18S rDNA.

### Leaf infiltration assays and analysis

*N*. *benthamiana* leaves (three leaves from three plants) were inoculated by infiltration with *Agrobacterium* cultures containing wild type or *cle-* mutant TGMV or CaLCuV DNA-A using a standard dose (OD_600_ = 1.0) as described [10,70]. DNA was isolated from discs cut from infiltration sites at 24, 48, and 72-hours post-inoculation (hpi). The presence of replicating viral DNA was assessed by DNA gel blot hybridization using the North2South Chemiluminescent Hybridization and Detection Kit (ThermoFisher Scientific) with HRP-labeled chemiluminescent probes specific for TGMV or CaLCuV DNA-A. Hybridization signals were detected and quantified by imager analysis using the C-Digit Blot Scanner (Licor Biosciences, Lincoln, NE).

To assess TCP transcript levels by transient assay, *Arabidopsis* seedlings were vacuum infiltrated with *Agrobacterium* cultures to deliver wild CaLCuV DNA-A as previously described [44]. Negative controls consisted of seedlings infiltrated with cultures containing the empty Ti plasmid vector (pMON521). For systemic response of TCP transcripts to CaLCuV, *Arabidopsis* plants were infected as previously described [4]. Symptomatic bolt tissue from virus infected plants or comparable uninfected tissue was harvested. RNA was extracted as detailed below and RT-qPCR was conducted using TCP specific primers. Significance was calculated using Student’s two tailed *t* test.

### RNA isolation and analysis

Total RNA was isolated from *N*. *benthamiana* leaves infiltrated with *Agrobacterium* containing wild type or *cle-* TGMV DNA 24, 48, and 72-hpi using Plant RNA Reagent (Invitrogen), followed by the removal of contaminating DNA by DNase treatment. The presence of TGMV CP mRNA was then assessed by generating cDNA from total RNA (500 ng) using a high-capacity cDNA archive kit (Applied Biosystems) and quantitative real-time PCR performed with primers specific for TGMV (TGMVqPCRNbF+ TGMVqPCRNbR) with SYBR green as described previously [66]. Subsequent analysis was performed with a QuantStudio 5 Real-time PCR system (ThermoFisher Scientific). For each experiment, the absolute amount of PCR product specific for the CP mRNA was determined using a standard curve generated from a PCR product specific for the CP ORF of TGMV. For each RNA sample, three leaf discs from each of three individual plants (nine plants total) were pooled prior to RNA isolation and three technical replicates used for the qPCR analysis. CP mRNA levels in *Arabidopsis* were determined as described [7]. Significance of differences in CP mRNA levels observed in symptomatic and asymptomatic *Arabidopsis* plants was assessed with Student’s *t*-test.

### Computational analysis

The TCP24 binding motif in the format of a position-specific probability matrix was obtained from Plant Transcription Factor Database (PlantTFDB) [72]. A small pseudocount of 0.001 was added to each probability and the matrix was then renormalized and converted to a position-specific weight matrix (PSWM): *W*_*ij*_ *= log*_*2*_ *((p*_*ij*_ *+ 0*.*001)/ 1*.*004)*, where *p*_*ij*_ is the probability of nucleotide *i* occurring in position *j* of the binding motif. Each promoter sequence and its reverse complementary sequence was then scanned with the PSWM for the subsequence that has the highest matching score. The matching score between a subsequence *S* and the weight matrix *W* is defined as *M(W*, *S)* = *Σ*_*j*_ *(W*_*Sj*,*j*_*)*, where *S*_*j*_ is the *j*-th nucleotide in *S*. To evaluate the statistical significance of a raw matching score, the promoter sequence was shuffled to obtain 1000 random sequences that have the same length as the real sequence. Each random sequence and its reverse complementary sequence were then scanned with the PSWM for the highest scoring match, similarly as for the real sequence. The raw matching score was converted to a p-value by a z-test using the mean and standard deviation of matching scores obtained from scanning the random sequences [73]. Finally, to correct for multiple testing, the p-values for all 92 promoter sequences (43 *BR1* and 49 *CP* promoter sequences) were converted to q-values using the Benjamini–Hochberg procedure [74], and a q-value less than 0.05 is considered statistically significant. The statistical significance of the number of bipartite begomoviruses having TCP binding site in both *CP* and *BR1* promoters was computed using the Fisher’s exact test [75].

## Supporting information

S1 Fig TCP24 interacts with the TGMV *CP* promoter in yeast. The target-reporter yeast strain, YM4271-TGMVCPactivator, was transformed with pGAD424-TCP24 (TCP) or the pGAD424 empty vector (GAD). The graphs represent growth of yeast strains at 30°C during a 24-hour period in liquid synthetic complete (SC) medium lacking histidine and leucine in the presence (90 mM) or absence (0 mM) of 3-aminotriazole (3-AT), as measured by optical density of the culture at 600 nm (OD 600nm).
(PDF)

S2 Fig Phylogenetic analysis of TCP proteins. *Arabidopsis* TCP transcription factors were compared to related proteins from *Solanum lycopersicum* (tomato) and *N*. *benthamiana* using available sequences (The Arabidopsis Resource Center, https://www.arabidopsis.org/ or SolGenomics Network, https://solgenomics.net/). Sequences were aligned using the M-Coffee sequence alignment tool (http://tcoffee.crg.cat/apps/tcoffee/do:mcoffee) [76]. A non-rooted tree was inferred using the Neighbor-Joining method. Percentages of replicate trees in which the associated taxa clustered together in the bootstrap test (1000 replicates) are shown next to the branches. TCP transcription factor domain structures are shown on the right. Conserved TCP (blue) and R domains (red), and the position of the miR319 recognition sequence in the mRNA (green) are indicated. Diagrams are not to scale. All TCPs contain a TCP domain, while the R domain is absent in all class I proteins and the class II CIN proteins (with some exceptions: AtTCP2, AtTCP24, NbTCP24, SlTCP24, SlTCP29). However, the R domain is present in most CYC/TB1 proteins. The target site for miR319 is only present in a subset of the CIN proteins (CIN-J).
(PDF)

S3 Fig Preparation of GST and 6xHis fusion proteins. (A) Western blot analysis of 6xHis-tagged AtTCP24 fusion protein expressed in *E*. *coli*. His-tagged TCP24 was purified using Ni-NTA agarose and detected using an anti-6xHis antibody as described in Methods. Presence of 6xHis-TCP24 (asterisk) in bound (B) and eluted (E1-3) fractions is shown. A 6xHis-AtPPD2 protein was used as a control for the antibody. (B) Western blot analysis of GST-tagged fusion proteins expressed in *N*. *benthamiana*. *Agrobacterium* containing DNA sequences capable of directing expression of GST or GST-tagged fusion proteins (GST-TCP24, GST-ERF13, and GST-AL2, control) from the TMV RNA-based (TRBO) vector [42] were used to infiltrate *N*. *benthamiana* leaves carrying a previously characterized A55M transgene [8]. Two days post-infiltration, leaves were ground in liquid nitrogen and total protein extracts analyzed by Western blot using an anti-GST antibody. Fusion proteins (asterisks) are indicated along the top with predicted molecular weights. (C) Western blot analysis of GST and GST-TCP24 expressed in *E*. *coli*. Proteins were induced and detected using an anti-GST antibody as described in Methods. Presence of GST and GST-TCP24 in induced (I) and soluble (SF) fractions is indicated by asterisks. NI, not induced.
(PDF)

S4 Fig TCP24 binding to proximal *CP* promoter sequences is not compromised by DNA containing distal repressor elements. 6xHis-TCP24 protein isolated from *E*. *coli* Rosetta cells (5 μg) was incubated with FITC-labeled DNA fragments containing the wild type (WT) TGMV or CaLCuV *CP* proximal promoter sequences in the presence (+) or absence (-) of a 50-fold molar excess of cold competitor DNA, as indicated. Complexes were separated on a 4–20% TBE gel. Positions of unbound probe (P) and TCP24 protein-probe DNA complexes (**) were detected by chemiluminescence. (A) TCP24-TGMV complexes (lane 2) were competed by excess DNA containing proximal TGMV *CP* promoter sequences (lane 3) but not by DNA containing TGMV or CaLCuV distal repressor elements (lanes 4 and 5). (B) Similarly, TCP24-CaLCuV complexes (lane 2) were competed by excess DNA containing proximal CaLCuV *CP* promoter sequences (lane 3) but not by DNA containing TGMV or CaLCuV distal repressor elements (lanes 4 and 5). Thus, distal repressor elements, which lack a CLE-like sequence, had no impact on binding.
(PDF)

S5 Fig Class II TCP binding sites (CLE) in *AR1/CP* and *BR1/NSP* promoters. Boxes are proportional to promoter length. Black vertical bars represent CLE positions, and orange bars indicate sites on the reverse complementary strand. Motif occurrence is determined if the matching score is significantly higher than in randomized sequences (q-value < 0.05, see Methods). CLE sequences are shown in purple. Sequences 400–500 bp upstream of the translation start sites were extracted from DNA-A and DNA-B of bipartite begomoviruses (black) African cassava mosaic virus (ACMV: NC_001467, NC_001468), bean calico mosaic virus (BCaMV: AF110189.1, AF110190), bean leaf crumple virus (BLCrV: KX857725, KX857726), Bhendi yellow vein mosaic virus (BYVMV: GU112079, HQ586007), cabbage leaf curl virus (CaLCuV: U65529, U65530), cotton leaf crumple virus (CLCrV: NC_004580.1, NC_004581), cucurbit leaf curl virus (CuLCuV: AF256200, AF327559), euphorbia yellow mosaic virus (EuYMV: FJ619507, FJ619508), macroptilium yellow mosaic virus (MacYMV: NC_010647, NC_010648), melon chlorotic leaf curl virus (MCLCuV: NC_004732, NC_028138), merremia mosaic virus (MerMV: AF068636, AY965899), pepper golden mosaic virus (PepGMV: AY928514, AY928515), pepper huasteco yellow vein virus (PHYVV: NC_001359, X70419), pepper mild tigré virus (PepMTV: EF210556.1, EF210557), pepper yellow leaf curl virus (PYLCuV: KX943290, KX943291), solanum mosaic Bolivia virus (SoMBoV: HM585435, HM585436), squash leaf curl virus (SLCuV: NC_001936, NC_001937), tomato dwarf leaf virus (ToDLV: NC_016580, NC_016581), tomato golden mosaic virus (TGMV: K02029, K02030), and tomato mottle leaf curl virus (ToMoLCuV: KX896412, MT214087). Sequences were also extracted from monopartite begomoviruses (teal) cotton leaf curl virus (CLCuV: FR819707), papaya leaf curl virus (PaLCuV: LT009399.), tomato yellow leaf curl virus (TYLCV: JQ867092), and curtoviruses (blue) spinach curly top virus (SCTV: AY548948) and beet curly top virus (BCTV: AF379637).
(PDF)

S6 Fig AtTCP24 localizes to the nucleus. Constructs expressing full length AtTCP24 or TGMV AL2 fused to YFP were delivered to *N*. *benthamiana* leaves by agroinfiltration. AL2 was previously shown to localize to the nucleus when fused to YFP [43]. Class II TCP proteins other than TCP24 have also been shown to localize to the nucleus [29]. Fluorescence was detected using a 40x objective with FITC (eGFP signal) and Rhodamine (RFP-Histone H4 signal) filter sets. Panels represent merged images from the two filter sets. The H4-RFP marker localizes to the nucleus [45], and is identified by red fluorescence (N). Green fluorescent signal can be clearly seen within the nuclei of cells expressing YFP-TGMV AL2 (N). In cells expressing YFP-TCP24, signal was mostly nuclear and often had a punctate appearance (N). Faint diffuse signal was sometimes also observed in the cytoplasm (C).
(PDF)

S7 Fig PCR analysis of chromatin used in ChIP assays. (A) Diagram of the A55M transgene, wherein the CP coding region is replaced by GUS and the AL2 gene is inactivated by mutation (AL2 X) [8]. Primer sets used in this experiment are indicated by arrows, and the approximate location of the distal repressor element (DR) is also indicated. (B) Chromatin was isolated from A55M transgenic *N*. *benthamiana* plants infiltrated with *Agrobacterium* containing DNA to express GST-tagged fusion proteins (GST-ERF13 or GST-TCP24) from the TRBO vector. Chromatin was isolated and sheared by sonication (shear size ~600 bp, see Methods) and total DNA isolated. Total DNA was also isolated from an aliquot of chromatin not subjected to sonication. PCR was performed on sheared (S) and unsheared (G) DNA using primer set 1 (CRA2F2 + GUSSeq, CP activator) to amplify the proximal *CP* promoter region and the first 200 bp of the GUS coding region (expected product 436 bp). Primer set 2 (CRA2F2 + GUSR red arrow, CP activator + GUS) was designed to amplify the *CP* promoter linked to the entire GUS coding sequence (expected size 2150 bp). A 436 bp product could be amplified both before (G) and after sonication (S) with primer set 1. However, using primer set 2, a 2150 bp PCR product was detectable only in total genomic DNA isolated from chromatin samples prior to sonication (G). Thus, the ChIP sonication protocol was sufficient to uncouple the proximal CP promoter from the distal repressor element. Marker represents fragments derived from a DNA ladder. NT = no template.
(PDF)

S8 Fig Systemic symptoms observed *in N*. *benthamiana* infected with wild type or *cle*- mutant viruses. The upper panels illustrate *N*. *benthamiana* plants infected with wild type (WT) or *cle*- mutant TGMV or CaLCuV 21 days post-inoculation. The lower panels are an enlarged view of systemic symptoms (red circles) caused by the different viruses.
(PDF)

S9 Fig CLE mutation in DNA-A decreases H3K27me3 levels on the IR of DNA-A, but not DNA-B. ChIP-qPCR experiments were performed with H3K27me3 antibody using nuclear extracts from *N*. *benthamiana* plants systemically infected with wild type (WT) or *cle-* CaLCuV DNA-A. In both cases, DNA-A was co-inoculated with WT DNA-B. Tissue from symptomatic plants was pooled for analysis. Data were normalized to input DNA, with signal from negative control IgG immunoprecipitate subtracted. Values were further normalized to ChIP-qPCRs performed with histone H3 antibody using the same extracts. (A) Locations of DNA-A component IR amplicons (A-E, ~100 bp) are illustrated in the diagram shown in Fig 11B. Three replicates are shown, with standard error of the mean. (B) The B-IR Right amplicon, which spans 152 bp and encompasses the WT CLE, was generated with a primer set specific for DNA-B. Two replicates are shown, with standard error of the mean.
(PDF)

S10 Fig *Arabidopsis clf-28* mutants infected with CaLCuV show attenuated symptoms. (A) Representative photo of mock inoculated or CaLCuV infected wild type (ecotype Col-0) or *clf-28* plants. Two plants are shown in each pot. White bars illustrate different degrees of stunting in CaLCuV infected plants. (B) Representation of the relative change in bolt height due to CaLCuV infection in wild type versus *clf-28* plants. Mock inoculated plant height was arbitrarily set to 1.0. Standard error of the mean is shown, and a two tailed Student’s t-test was used to test significance. (C) CaLCuV DNA levels. Total DNA was extracted, and viral DNA measured by qPCR using primers specific for the intergenic region of DNA A or DNA B. Viral DNA was first normalized to 18S DNA and then levels of DNA A or DNA B in *clf-28* plants expressed as a fold change relative to DNA levels in wild type (ecotype Col-0). The number of copies of DNA-A and DNA-B were similar in wild type plants (DNA-A, 7.0–7.6 copies/μl sample; DNA-B, 7.5–8.5 copies/μl sample) and *clf-28* plants (DNA-A, 6.8–7.4 copies/μl sample; DNA-B, 7.4–8.0 copies/μl sample). (D) CP mRNA levels. Total RNA was extracted, and CP mRNA measured by RT-qPCR using primers specific for the CP coding region. Viral RNA was normalized to total viral DNA as measured by qPCR, then normalized to PP2A.
(PDF)

S1 Table Viral DNA loads in *N*. *benthamiana* plants inoculated with TGMV wild type or *cle*- mutant viruses. (PDF)

S2 Table Viral DNA loads in *N*. *benthamiana* plants inoculated with CaLCuV wild type or *cle-* mutant viruses. (PDF)

S3 Table Time course of viral DNA loads in *N*. *benthamiana* plants inoculated with TGMV or CaLCuV wild type or *cle-* DNA-A. (PDF)

S4 Table Time course of viral CP mRNA levels in *N*. *benthamiana* plants inoculated with TGMV wild type or *cle-* DNA-A. (PDF)

S5 Table Primers used in this study for cloning, PCR, and qPCR. (PDF)
