# Supplementary figures and images for: Mutation of the conserved late element in geminivirus CP promoters abolishes Arabidopsis TCP24 transcription factor binding and decreases H3K27me3 levels on viral chromatin
Source: PLoS Pathog. 2024 Jul 18;20(7):e1012399. doi: 10.1371/journal.ppat.1012399 (PMC11288445; doi:10.1371/journal.ppat.1012399)

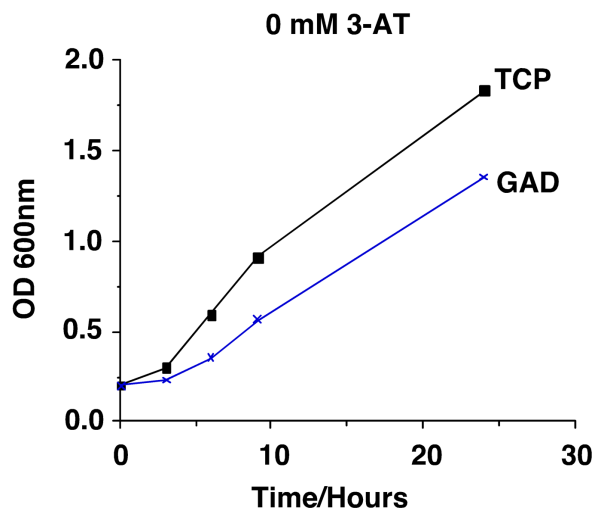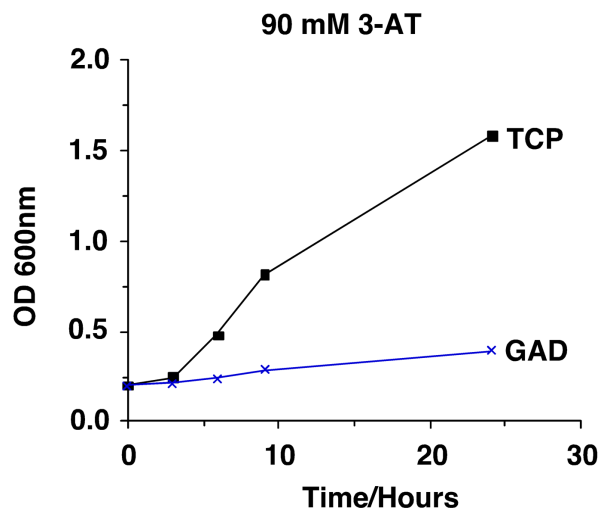

Supplement: S1 Fig — The target-reporter yeast strain, YM4271-TGMVCPactivator, was transformed with pGAD424-TCP24 (TCP) or the pGAD424 empty vector (GAD). The graphs represent growth of yeast strains at 30°C during a 24-hour period in liquid synthetic complete (SC) medium lacking histidine and leucine in the presence (90 mM) or absence (0 mM) of 3-aminotriazole (3-AT), as measured by optical density of the culture at 600 nm (OD 600nm). (PDF) [file ppat.1012399.s001.pdf]

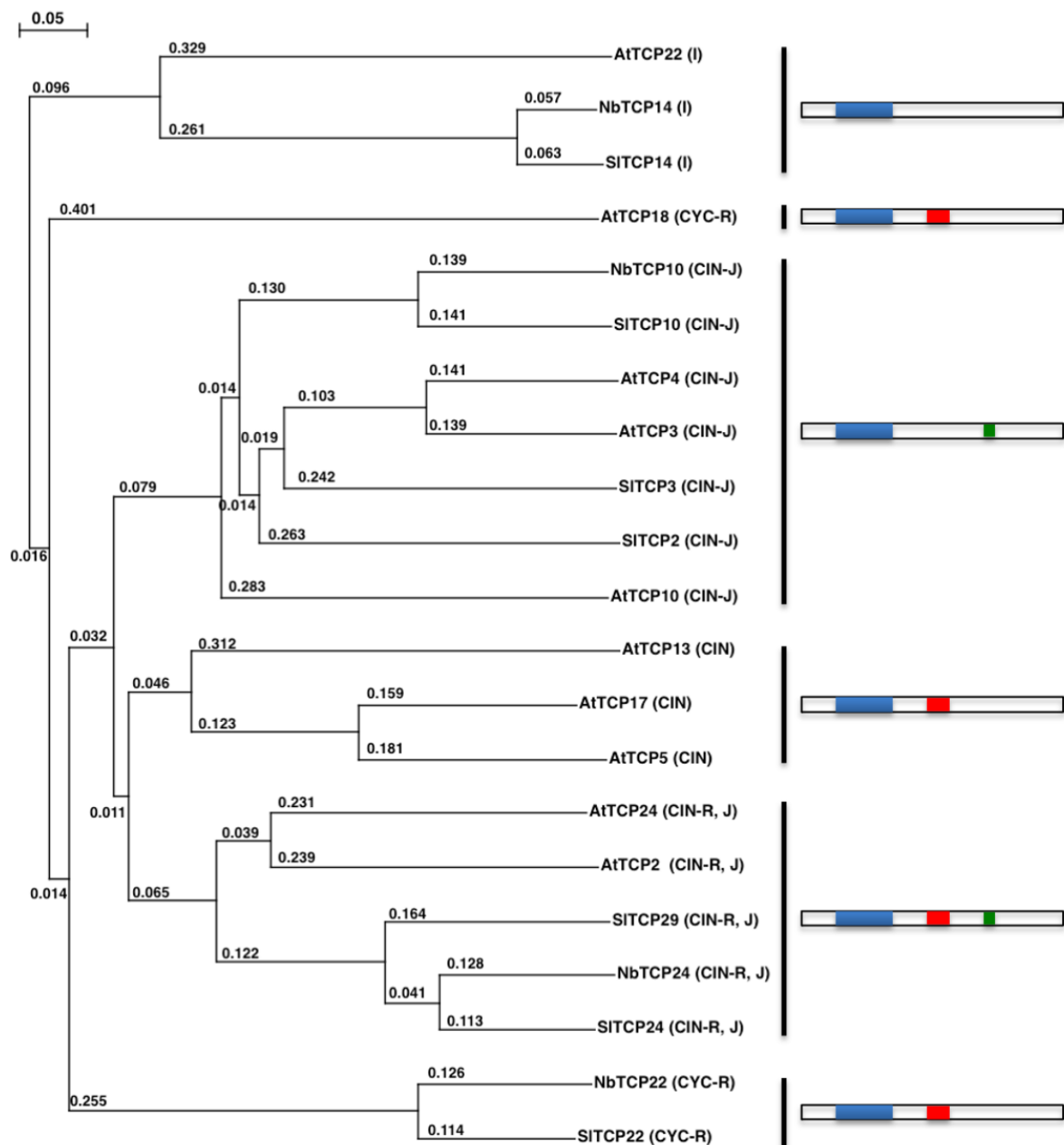

Supplement: S2 Fig — Arabidopsis TCP transcription factors were compared to related proteins from Solanum lycopersicum (tomato) and N. benthamiana using available sequences (The Arabidopsis Resource Center, https://www.arabidopsis.org/ or SolGenomics Network, https://solgenomics.net/). Sequences were aligned using the M-Coffee sequence alignment tool (http://tcoffee.crg.cat/apps/tcoffee/do:mcoffee) [76]. A non-rooted tree was inferred using the Neighbor-Joining method. Percentages of replicate trees in which the associated taxa clustered together in the bootstrap test (1000 replicates) are shown next to the branches. TCP transcription factor domain structures are shown on the right. Conserved TCP (blue) and R domains (red), and the position of the miR319 recognition sequence in the mRNA (green) are indicated. Diagrams are not to scale. All TCPs contain a TCP domain, while the R domain is absent in all class I proteins and the class II CIN proteins (with some exceptions: AtTCP2, AtTCP24, NbTCP24, SlTCP24, SlTCP29). However, the R domain is present in most CYC/TB1 proteins. The target site for miR319 is only present in a subset of the CIN proteins (CIN-J). (PDF) [file ppat.1012399.s002.pdf]

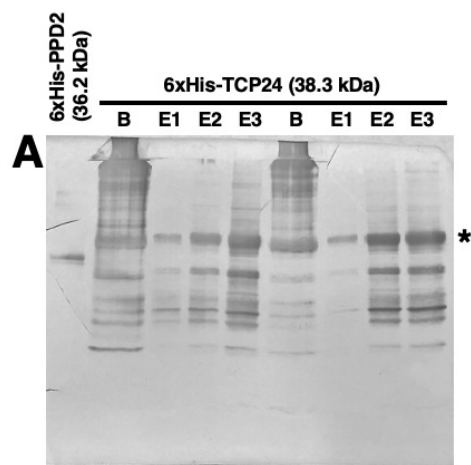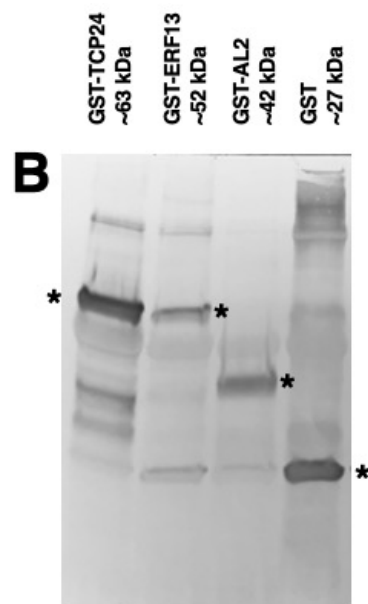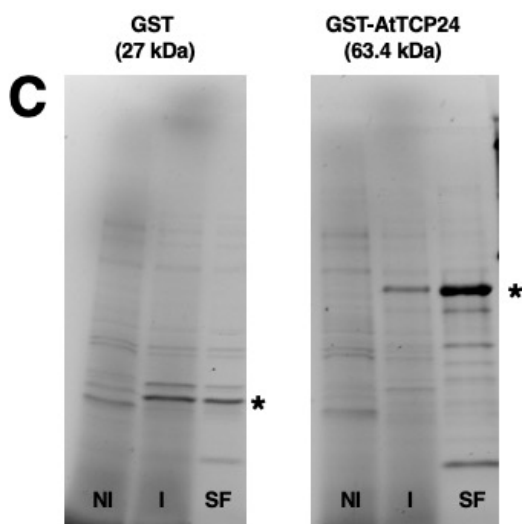

Supplement: S3 Fig — (A) Western blot analysis of 6xHis-tagged AtTCP24 fusion protein expressed in E. coli. His-tagged TCP24 was purified using Ni-NTA agarose and detected using an anti-6xHis antibody as described in Methods. Presence of 6xHis-TCP24 (asterisk) in bound (B) and eluted (E1-3) fractions is shown. A 6xHis-AtPPD2 protein was used as a control for the antibody. (B) Western blot analysis of GST-tagged fusion proteins expressed in N. benthamiana. Agrobacterium containing DNA sequences capable of directing expression of GST or GST-tagged fusion proteins (GST-TCP24, GST-ERF13, and GST-AL2, control) from the TMV RNA-based (TRBO) vector [42] were used to infiltrate N. benthamiana leaves carrying a previously characterized A55M transgene [8]. Two days post-infiltration, leaves were ground in liquid nitrogen and total protein extracts analyzed by Western blot using an anti-GST antibody. Fusion proteins (asterisks) are indicated along the top with predicted molecular weights. (C) Western blot analysis of GST and GST-TCP24 expressed in E. coli. Proteins were induced and detected using an anti-GST antibody as described in Methods. Presence of GST and GST-TCP24 in induced (I) and soluble (SF) fractions is indicated by asterisks. NI, not induced. (PDF) [file ppat.1012399.s003.pdf]

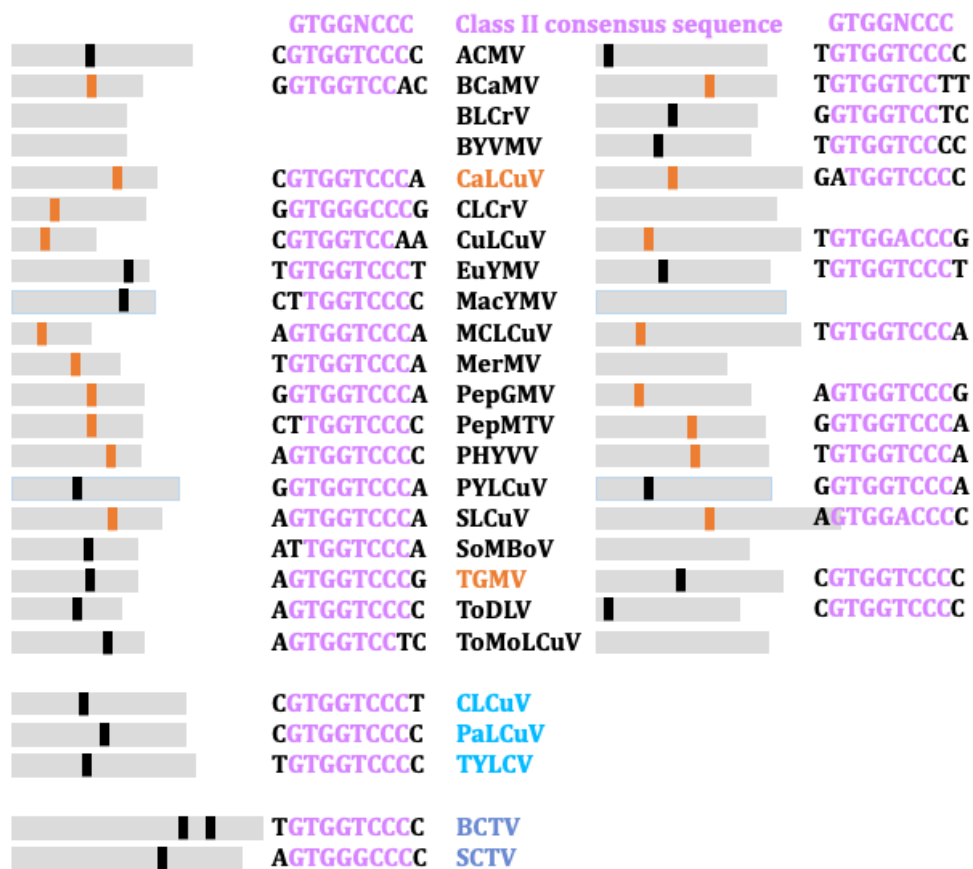

TCP Motif in AR1 Promoter

TCP Motif in BR1 Promoter

Supplement: S5 Fig — Boxes are proportional to promoter length. Black vertical bars represent CLE positions, and orange bars indicate sites on the reverse complementary strand. Motif occurrence is determined if the matching score is significantly higher than in randomized sequences (q-value < 0.05, see Methods). CLE sequences are shown in purple. Sequences 400–500 bp upstream of the translation start sites were extracted from DNA-A and DNA-B of bipartite begomoviruses (black) African cassava mosaic virus (ACMV: NC_001467, NC_001468), bean calico mosaic virus (BCaMV: AF110189.1, AF110190), bean leaf crumple virus (BLCrV: KX857725, KX857726), Bhendi yellow vein mosaic virus (BYVMV: GU112079, HQ586007), cabbage leaf curl virus (CaLCuV: U65529, U65530), cotton leaf crumple virus (CLCrV: NC_004580.1, NC_004581), cucurbit leaf curl virus (CuLCuV: AF256200, AF327559), euphorbia yellow mosaic virus (EuYMV: FJ619507, FJ619508), macroptilium yellow mosaic virus (MacYMV: NC_010647, NC_010648), melon chlorotic leaf curl virus (MCLCuV: NC_004732, NC_028138), merremia mosaic virus (MerMV: AF068636, AY965899), pepper golden mosaic virus (PepGMV: AY928514, AY928515), pepper huasteco yellow vein virus (PHYVV: NC_001359, X70419), pepper mild tigré virus (PepMTV: EF210556.1, EF210557), pepper yellow leaf curl virus (PYLCuV: KX943290, KX943291), solanum mosaic Bolivia virus (SoMBoV: HM585435, HM585436), squash leaf curl virus (SLCuV: NC_001936, NC_001937), tomato dwarf leaf virus (ToDLV: NC_016580, NC_016581), tomato golden mosaic virus (TGMV: K02029, K02030), and tomato mottle leaf curl virus (ToMoLCuV: KX896412, MT214087). Sequences were also extracted from monopartite begomoviruses (teal) cotton leaf curl virus (CLCuV: FR819707), papaya leaf curl virus (PaLCuV: LT009399.), tomato yellow leaf curl virus (TYLCV: JQ867092), and curtoviruses (blue) spinach curly top virus (SCTV: AY548948) and beet curly top virus (BCTV: AF379637). (PDF) [file ppat.1012399.s005.pdf]

**YFP-AtTCP24**

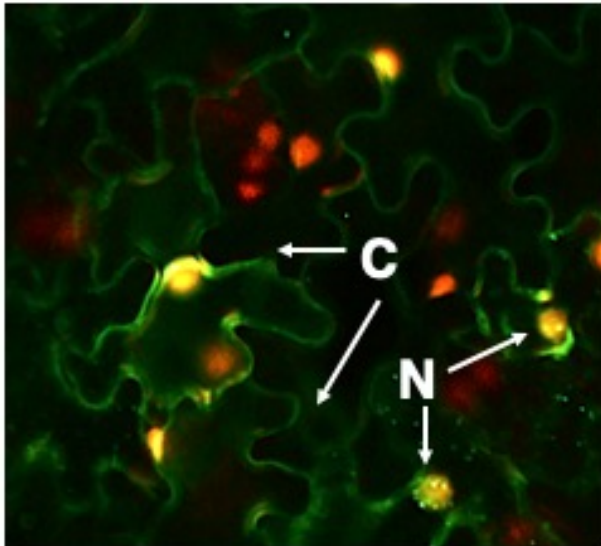

**YFP-TGMV AL2**

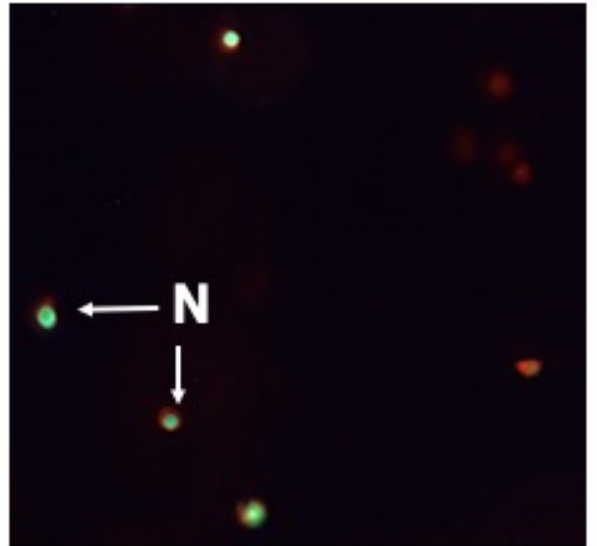

Supplement: S6 Fig — Constructs expressing full length AtTCP24 or TGMV AL2 fused to YFP were delivered to N. benthamiana leaves by agroinfiltration. AL2 was previously shown to localize to the nucleus when fused to YFP [43]. Class II TCP proteins other than TCP24 have also been shown to localize to the nucleus [29]. Fluorescence was detected using a 40x objective with FITC (eGFP signal) and Rhodamine (RFP-Histone H4 signal) filter sets. Panels represent merged images from the two filter sets. The H4-RFP marker localizes to the nucleus [45], and is identified by red fluorescence (N). Green fluorescent signal can be clearly seen within the nuclei of cells expressing YFP-TGMV AL2 (N). In cells expressing YFP-TCP24, signal was mostly nuclear and often had a punctate appearance (N). Faint diffuse signal was sometimes also observed in the cytoplasm (C). (PDF) [file ppat.1012399.s006.pdf]

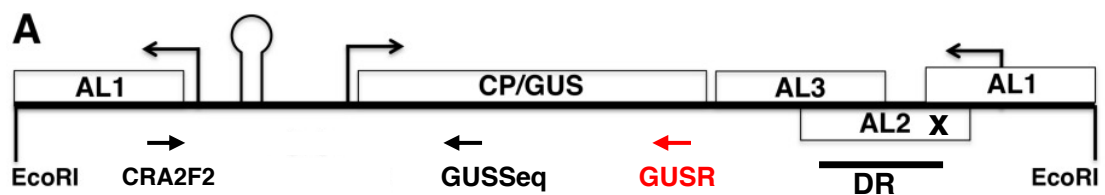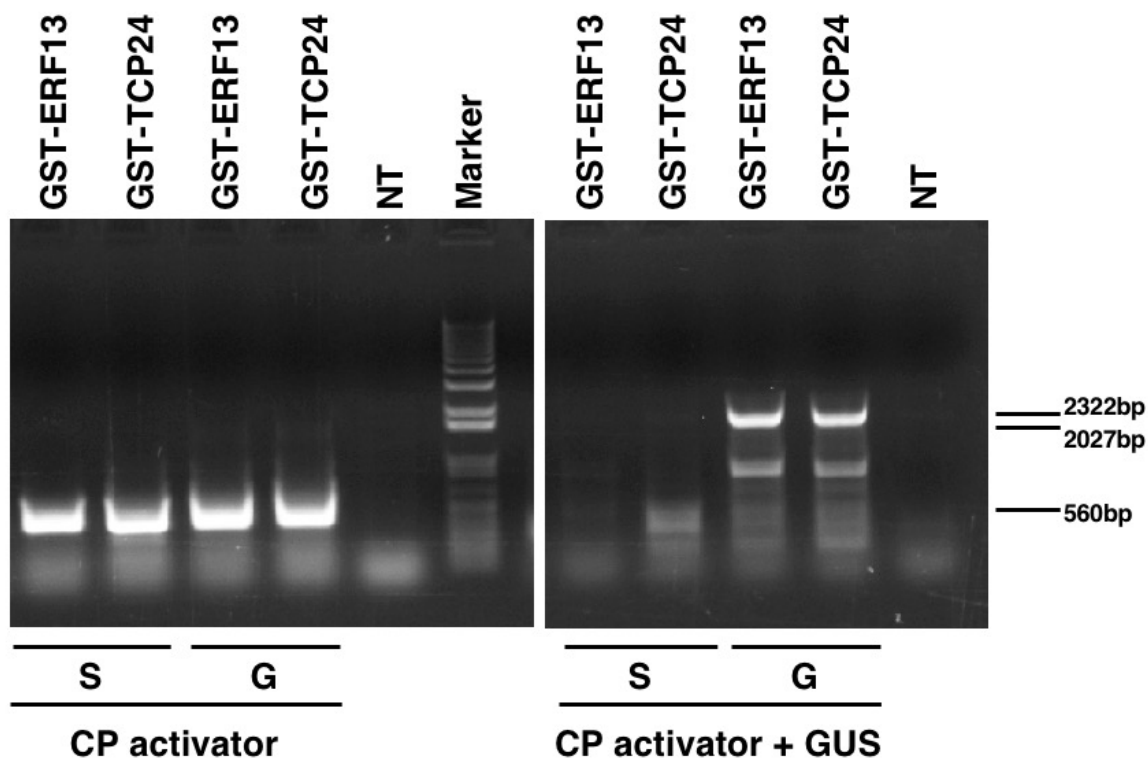

Supplement: S7 Fig — (A) Diagram of the A55M transgene, wherein the CP coding region is replaced by GUS and the AL2 gene is inactivated by mutation (AL2 X) [8]. Primer sets used in this experiment are indicated by arrows, and the approximate location of the distal repressor element (DR) is also indicated. (B) Chromatin was isolated from A55M transgenic N. benthamiana plants infiltrated with Agrobacterium containing DNA to express GST-tagged fusion proteins (GST-ERF13 or GST-TCP24) from the TRBO vector. Chromatin was isolated and sheared by sonication (shear size ~600 bp, see Methods) and total DNA isolated. Total DNA was also isolated from an aliquot of chromatin not subjected to sonication. PCR was performed on sheared (S) and unsheared (G) DNA using primer set 1 (CRA2F2 + GUSSeq, CP activator) to amplify the proximal CP promoter region and the first 200 bp of the GUS coding region (expected product 436 bp). Primer set 2 (CRA2F2 + GUSR red arrow, CP activator + GUS) was designed to amplify the CP promoter linked to the entire GUS coding sequence (expected size 2150 bp). A 436 bp product could be amplified both before (G) and after sonication (S) with primer set 1. However, using primer set 2, a 2150 bp PCR product was detectable only in total genomic DNA isolated from chromatin samples prior to sonication (G). Thus, the ChIP sonication protocol was sufficient to uncouple the proximal CP promoter from the distal repressor element. Marker represents fragments derived from a DNA ladder. NT = no template. (PDF) [file ppat.1012399.s007.pdf]

# TGMV

WT

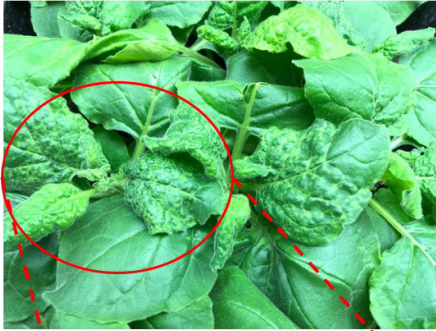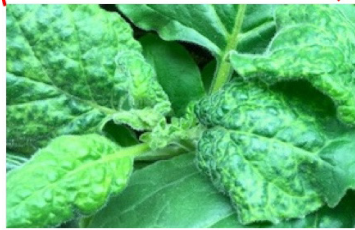

*cle-*

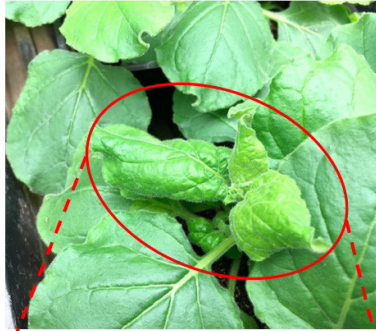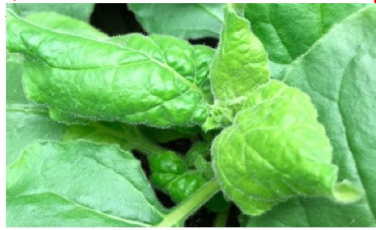

# CaLCuV

WT

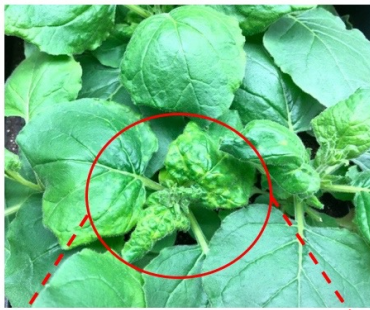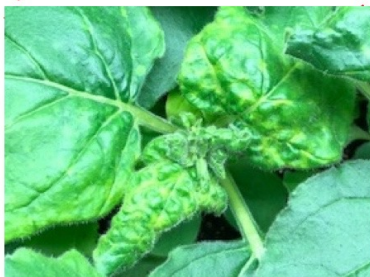

*cle-*

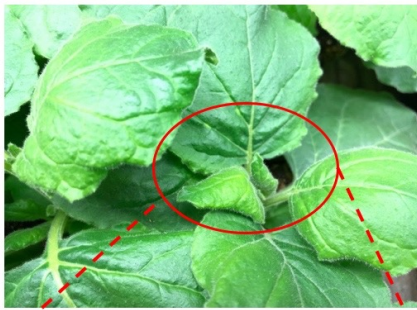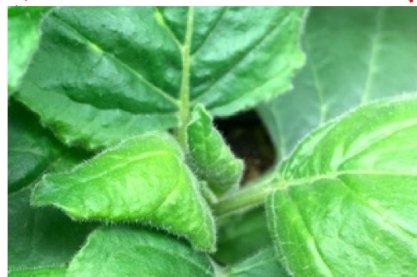

Supplement: S8 Fig — The upper panels illustrate N. benthamiana plants infected with wild type (WT) or cle- mutant TGMV or CaLCuV 21 days post-inoculation. The lower panels are an enlarged view of systemic symptoms (red circles) caused by the different viruses. (PDF) [file ppat.1012399.s008.pdf]

**A**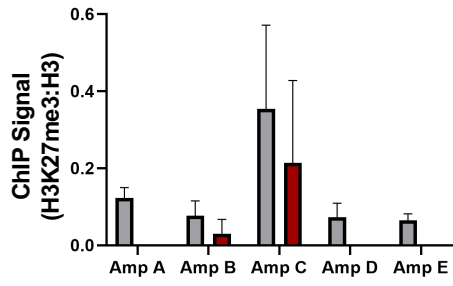**B**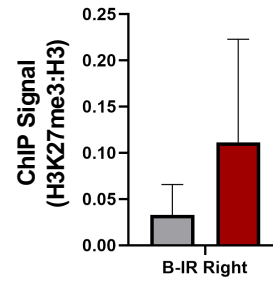

Supplement: S9 Fig — ChIP-qPCR experiments were performed with H3K27me3 antibody using nuclear extracts from N. benthamiana plants systemically infected with wild type (WT) or cle- CaLCuV DNA-A. In both cases, DNA-A was co-inoculated with WT DNA-B. Tissue from symptomatic plants was pooled for analysis. Data were normalized to input DNA, with signal from negative control IgG immunoprecipitate subtracted. Values were further normalized to ChIP-qPCRs performed with histone H3 antibody using the same extracts. (A) Locations of DNA-A component IR amplicons (A-E, ~100 bp) are illustrated in the diagram shown in Fig 11B. Three replicates are shown, with standard error of the mean. (B) The B-IR Right amplicon, which spans 152 bp and encompasses the WT CLE, was generated with a primer set specific for DNA-B. Two replicates are shown, with standard error of the mean. (PDF) [file ppat.1012399.s009.pdf]

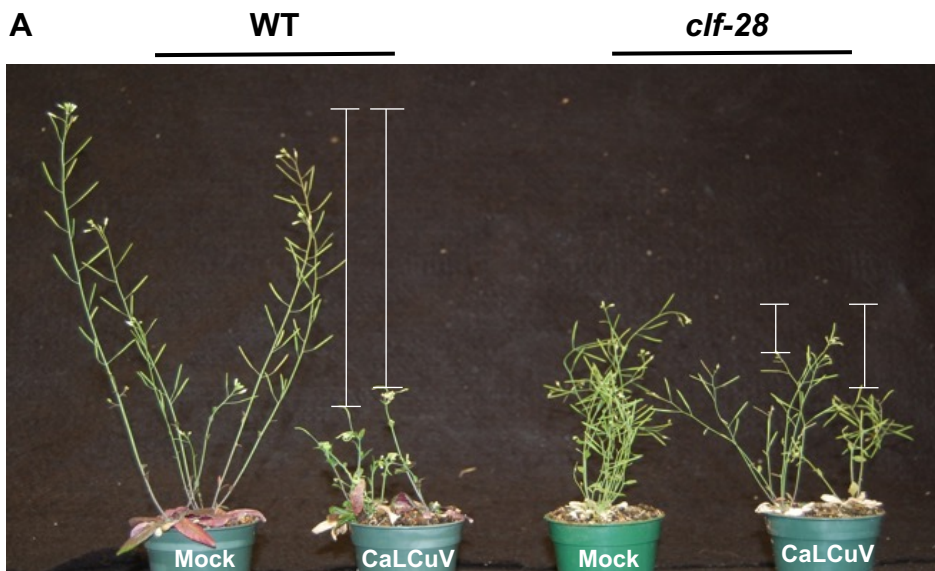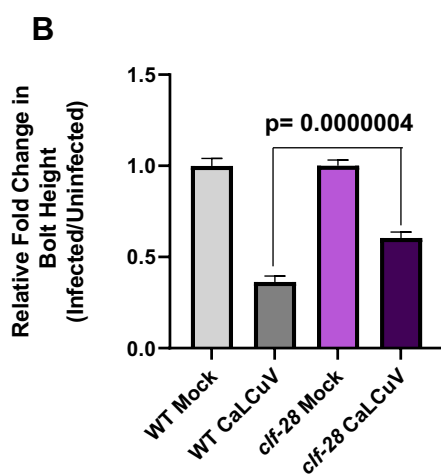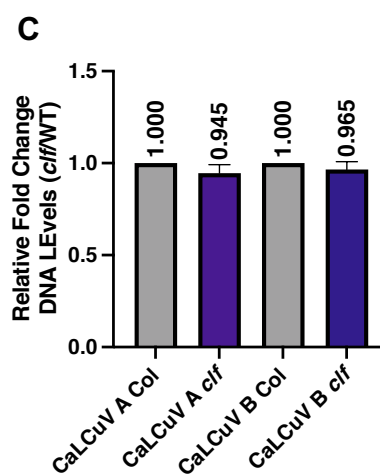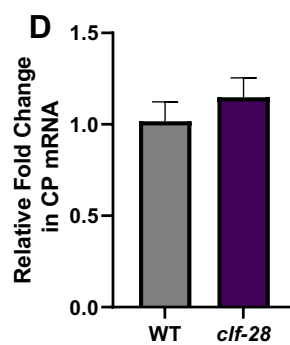

Supplement: S10 Fig — (A) Representative photo of mock inoculated or CaLCuV infected wild type (ecotype Col-0) or clf-28 plants. Two plants are shown in each pot. White bars illustrate different degrees of stunting in CaLCuV infected plants. (B) Representation of the relative change in bolt height due to CaLCuV infection in wild type versus clf-28 plants. Mock inoculated plant height was arbitrarily set to 1.0. Standard error of the mean is shown, and a two tailed Student’s t-test was used to test significance. (C) CaLCuV DNA levels. Total DNA was extracted, and viral DNA measured by qPCR using primers specific for the intergenic region of DNA A or DNA B. Viral DNA was first normalized to 18S DNA and then levels of DNA A or DNA B in clf-28 plants expressed as a fold change relative to DNA levels in wild type (ecotype Col-0). The number of copies of DNA-A and DNA-B were similar in wild type plants (DNA-A, 7.0–7.6 copies/μl sample; DNA-B, 7.5–8.5 copies/μl sample) and clf-28 plants (DNA-A, 6.8–7.4 copies/μl sample; DNA-B, 7.4–8.0 copies/μl sample). (D) CP mRNA levels. Total RNA was extracted, and CP mRNA measured by RT-qPCR using primers specific for the CP coding region. Viral RNA was normalized to total viral DNA as measured by qPCR, then normalized to PP2A. (PDF) [file ppat.1012399.s010.pdf]
