# Supplementary material for: Mutation of the conserved late element in geminivirus CP promoters abolishes Arabidopsis TCP24 transcription factor binding and decreases H3K27me3 levels on viral chromatin
Source: PLoS Pathog. 2024 Jul 18;20(7):e1012399. doi: 10.1371/journal.ppat.1012399 (PMC11288445; doi:10.1371/journal.ppat.1012399)
Supplement: S4 Fig — 6xHis-TCP24 protein isolated from E. coli Rosetta cells (5 μg) was incubated with FITC-labeled DNA fragments containing the wild type (WT) TGMV or CaLCuV CP proximal promoter sequences in the presence (+) or absence (-) of a 50-fold molar excess of cold competitor DNA, as indicated. Complexes were separated on a 4–20% TBE gel. Positions of unbound probe (P) and TCP24 protein-probe DNA complexes (**) were detected by chemiluminescence. (A) TCP24-TGMV complexes (lane 2) were competed by excess DNA containing proximal TGMV CP promoter sequences (lane 3) but not by DNA containing TGMV or CaLCuV distal repressor elements (lanes 4 and 5). (B) Similarly, TCP24-CaLCuV complexes (lane 2) were competed by excess DNA containing proximal CaLCuV CP promoter sequences (lane 3) but not by DNA containing TGMV or CaLCuV distal repressor elements (lanes 4 and 5). Thus, distal repressor elements, which lack a CLE-like sequence, had no impact on binding. (PDF) [file ppat.1012399.s004.pdf]

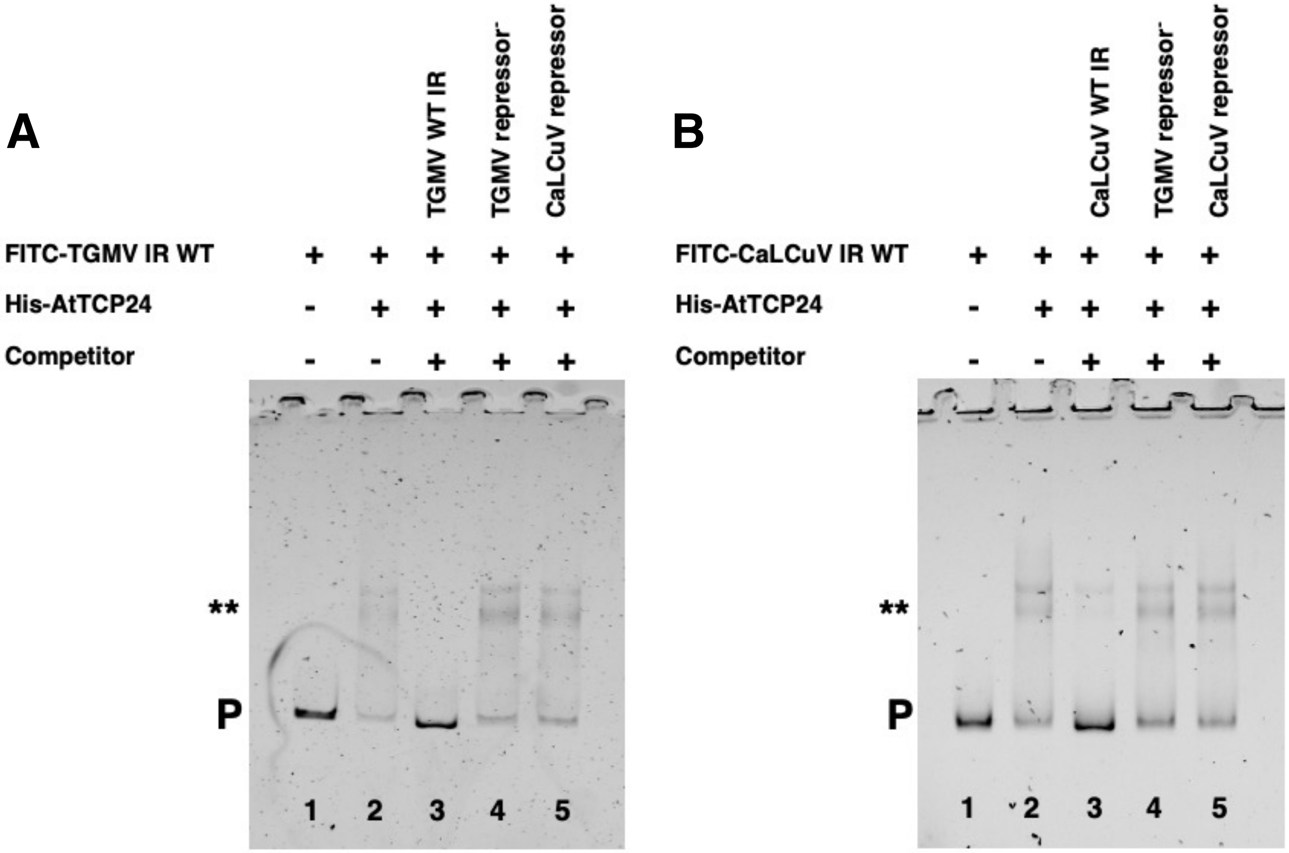
A
TGMV WT IR
TGMV repressor⁻
CaLCuV repressor
FITC-TGMV IR WT + + + + +
His-AtTCP24 - + + + +
Competitor - - + + +
**
P
1 2 3 4 5
B
CaLCuV WT IR
TGMV repressor⁻
CaLCuV repressor
FITC-CaLCuV IR WT + + + + +
His-AtTCP24 - + + + +
Competitor - - + + +
**
P
1 2 3 4 5
