# Supplementary material for: Mutation of the conserved late element in geminivirus CP promoters abolishes Arabidopsis TCP24 transcription factor binding and decreases H3K27me3 levels on viral chromatin
Source: PLoS Pathog. 2024 Jul 18;20(7):e1012399. doi: 10.1371/journal.ppat.1012399 (PMC11288445; doi:10.1371/journal.ppat.1012399)
Supplement: S1 Table — (PDF) [file ppat.1012399.s011.pdf]

S1 Table. Viral DNA loads in *N. benthamiana* plants inoculated with TGMV wild type or *cle-* mutant viruses.

Individual Plant Samples[1]	ng Viral DNA[2]	Viral Copy #[4]
TGMV wt S	127	4.5×10^{10}
TGMV wt S	157	5.6×10^{10}
TGMV wt S	141	5.0×10^{10}
TGMV wt S	72	2.6×10^{10}
TGMV wt S	239	8.6×10^{10}
TGMV wt S	166	5.9×10^{10}
**Mean (± SEM)**	**150 ± 22**	**$5.4 \times 10^{10} \pm 0.8 \times 10^{10}$**
TGMV *cle-* AS	6.6×10^{-8}	2.4×10^{1}
TGMV *cle-* AS	2.2×10^{-7}	7.8×10^{1}
TGMV *cle-* AS	7.7×10^{-7}	2.8×10^{2}
TGMV *cle-* AS	1.9×10^{-8}	6.8×10^{0}
TGMV *cle-* AS	1.3×10^{-8}	4.7×10^{0}
TGMV *cle-* AS	1.8×10^{-7}	6.4×10^{1}
**Mean (± SEM)**	**$2.1 \times 10^{-7} \pm 1.2 \times 10^{-7}$**	**$7.5 \times 10^{1} \pm 4.2 \times 10^{1}$**
TGMV *cle-* S	6.8	2.4×10^{9}
TGMV *cle-* S	3.6	1.3×10^{9}
TGMV *cle-* S	0.8	2.7×10^{8}
TGMV *cle-* S	0.2	7.5×10^{6}
TGMV *cle-* S**	1.9×10^{-7}	6.9×10^{1}
TGMV *cle-* S**	8.2×10^{-8}	2.9×10^{1}
**Mean (± SEM)**	**$2.8 \times 10^{1} \pm 1.1 \times 10^{1}$**	**$6.7 \times 10^{8} \pm 4.1 \times 10^{8}$**

[1]Individual plants that were either symptomatic (S) or asymptomatic (AS) after inoculation with TGMV DNA-A containing a wild type (wt) or mutated (*cle-*) class II TCP binding site, and wild type DNA-B.
[2]The amount (ng) of viral DNA present in tissue isolated from individual plants as determined by comparison to a standard curve in qPCR reactions. The mean amount of viral DNA ± the standard error of the mean (SEM) calculated using the values for individual plants is given in bold.
[3]Number of copies of viral DNA present in tissue isolated from individual plants. The mean viral DNA copy number ± the standard error of the mean (SEM) calculated using the values for individual plants is given in bold.
**Plants infected with TGMV DNA-A *cle-* that exhibited very mild symptoms could have been scored as asymptomatic based on viral DNA loads
