# Supplementary material for: Mutation of the conserved late element in geminivirus CP promoters abolishes Arabidopsis TCP24 transcription factor binding and decreases H3K27me3 levels on viral chromatin
Source: PLoS Pathog. 2024 Jul 18;20(7):e1012399. doi: 10.1371/journal.ppat.1012399 (PMC11288445; doi:10.1371/journal.ppat.1012399)
Supplement: S2 Table — (PDF) [file ppat.1012399.s012.pdf]

S2 Table. Viral DNA loads in *N. benthamiana* plants inoculated with CaLCuV wild type or *cle-* mutant viruses.

Individual Plant Samples[1]	ng Viral DNA[2]	Viral Copy #[4]
CaLCuV wt S	5.9	2.1×10^{9}
CaLCuV wt S	10.5	3.8×10^{9}
CaLCuV wt S	10.3	3.7×10^{9}
CaLCuV wt S	15.0	5.4×10^{9}
CaLCuV wt S	6.5	2.3×10^{9}
CaLCuV wt S	8.1	2.9×10^{9}
**Mean (± SEM)**	**9.4 ± 1.4**	**$3.4 \times 10^{9} \pm 0.5 \times 10^{9}$**
CaLCuV *cle-* AS	1.5×10^{-4}	5.3×10^{4}
CaLCuV *cle-* AS	3.2×10^{-5}	1.1×10^{4}
CaLCuV *cle-* AS	3.7×10^{-6}	1.3×10^{3}
CaLCuV *cle-* AS	1.0×10^{-5}	3.6×10^{3}
CaLCuV *cle-* AS	2.3×10^{-5}	8.3×10^{3}
CaLCuV *cle-* AS	3.6×10^{-4}	1.3×10^{5}
**Mean (± SEM)**	**$9.6 \times 10^{-5} \pm 3.9 \times 10^{-5}$**	**$3.5 \times 10^{4} \pm 2.1 \times 10^{4}$**
CaLCuV *cle-* S	8.3×10^{-3}	3.0×10^{6}
CaLCuV *cle-* S	3.7×10^{-2}	1.3×10^{7}
CaLCuV *cle-* S	1.2×10^{-1}	4.3×10^{7}
CaLCuV *cle-* S	2.3×10^{-2}	8.1×10^{6}
CaLCuV *cle-* S	9.4×10^{-2}	3.4×10^{7}
CaLCuV *cle-* S	1.7×10^{-1}	6.1×10^{7}
**Mean (± SEM)**	**$7.5 \times 10^{-2} \pm 2.6 \times 10^{-2}$**	**$3.2 \times 10^{7} \pm 0.9 \times 10^{7}$**

[1]Individual plants that were either symptomatic (S) or asymptomatic (AS) after inoculation with CaLCuV DNA-A containing a wild type (wt) or mutated (*cle-*) class II TCP binding site, and wild type DNA-B.
[2]The amount of viral DNA in ng present in tissue isolated from individual plants as determined by comparison to a standard curve in qPCR reactions. The mean amount of viral DNA ± the standard error of the mean (SEM) calculated using the values for individual plants is given in bold.
[3]Number of copies of viral DNA present in tissue isolated from individual plants. The mean viral DNA copy number ± the standard error of the mean (SEM) calculated using the values for individual plants is given in bold.
