# Supplementary material for: Mutation of the conserved late element in geminivirus CP promoters abolishes Arabidopsis TCP24 transcription factor binding and decreases H3K27me3 levels on viral chromatin
Source: PLoS Pathog. 2024 Jul 18;20(7):e1012399. doi: 10.1371/journal.ppat.1012399 (PMC11288445; doi:10.1371/journal.ppat.1012399)
Supplement: S3 Table — (PDF) [file ppat.1012399.s013.pdf]

S3 Table Time course of viral DNA loads in *N. benthamiana* plants inoculated with TGMV or CaLCuV wild type or *cle-* DNA A.

hpi[1]	Virus[2]	DNA Form[3]	Signal[4]	dsDNA[5]	ssDNA	Total[6]
48	TGMV wt	lin	0			
		oc	11377			
		ccc	64879	76256		
		ss	81609		81609	157865
48	TGMV *cle-*	lin	35632			
		oc	0			
		ccc	43550	79182		
		ss	0		0	79182
72	TGMV wt	lin	50258			
		oc	35490			
		ccc	175154	260901		
		ss	97603		97603	358504
72	TGMV *cle-*	lin	177761			
		oc	0			
		ccc	221988	399749		
		ss	0		0	399749
48	CaLCuV wt	lin	9748			
		oc	26829			
		ccc	27556	64134		
		ss	17436		17436	81569
48	CaLCuV *cle-*	lin	11393			
		oc	0			
		ccc	12062	23456		
		ss	5403		5403	28858
72	CaLCuV wt	lin	36935			
		oc	16379			
		ccc	36320	89634		
		ss	17005		17005	106640
72	CaLCuV *cle-*	lin	53936			
		oc	23008			
		ccc	62481	106640		
		ss	29170		29170	168595

[1]The time in hours that total DNA was isolated from *N. benthamiana* leaves after infiltration

[2]*N. benthamiana* leaves were infused with agrobacterium containing either wild type (wt) or mutant (*cle*-) TGMV DNA A or CaLCuV DNA A.

[3]Replicating DNA forms detectable in total DNA samples isolated from *N. benthamiana* leaves. Lin – linear; oc – open circular; ccc – covalently closed circular; ss – single stranded.

[4]Chemiluminescence signal for each replicating DNA form detected on Southern Blots using a C-Digit Blot Scanner (Licor).

[5]Total chemiluminscence signal for dsDNA forms detected (lin + oc + ccc)

[6]Total chemiluminscence signal for all DNA forms detected ((lin + oc + ccc + ss).
