# Supplementary material for: Mutation of the conserved late element in geminivirus CP promoters abolishes Arabidopsis TCP24 transcription factor binding and decreases H3K27me3 levels on viral chromatin
Source: PLoS Pathog. 2024 Jul 18;20(7):e1012399. doi: 10.1371/journal.ppat.1012399 (PMC11288445; doi:10.1371/journal.ppat.1012399)
Supplement: S4 Table — (PDF) [file ppat.1012399.s014.pdf]

S4 Table. Time course of viral CP mRNA levels in *N. benthamiana* plants inoculated with TGMV wild type or *cle-* DNA-A.

hpi[1]	Virus[2]	CP mRNA Copy #[3]	Relative dsDNA Levels[4]	CP Copy #[5] /dsDNA
24	TGMV wt	4.7×10^5	NC	4.7×10^5
24	TGMV *cle-*	0.5×10^5	NC	0.5×10^5
48	TGMV wt	29×10^5	1.00	29×10^5
48	TGMV *cle-*	14×10^5	1.04	13×10^5
72	TGMV wt	13×10^5	1.00	13×10^5
72	TGMV *cle-*	3.4×10^5	1.53	2.2×10^5

[1]The time in hours that total DNA was isolated from *N. benthamiana* leaves after infiltration.
[2]*N. benthamiana* leaves were infiltrated with *Agrobacterium* containing either wild type (wt) or mutant (*cle*) TGMV DNA-A.
[3]Absolute levels of CP mRNA levels in 500ng total RNA isolated from *N. benthamiana* leaves were calculated by qPCR using a standard curve.
[4]Relative levels of dsDNA template were calculated using the data from S4 Table. NC = not calculated.
[5]CP mRNA levels were adjusted for the relative amounts of dsDNA template for 48- and 72-hpi as data for 24-hpi was not calcuated.
