# Supplementary material for: Mutation of the conserved late element in geminivirus CP promoters abolishes Arabidopsis TCP24 transcription factor binding and decreases H3K27me3 levels on viral chromatin
Source: PLoS Pathog. 2024 Jul 18;20(7):e1012399. doi: 10.1371/journal.ppat.1012399 (PMC11288445; doi:10.1371/journal.ppat.1012399)
Supplement: S5 Table — (PDF) [file ppat.1012399.s015.pdf]

Table S5. Primers used in this study for cloning, PCR and qPCR

Primer Name	Primer Sequences
AtTCP24entrF	5'-CACCATGGAGGTTGACGAAGAC-3'
AtTCP24entrR	5'-CTATCTCCTTTCCTTTGC-3'
AtTCP24entrLinkerF	5'-CACCATGGCTGCAGCTGCAGCTGCAATGGAGGTTGACGAAGAC-3'
AtTCP24SnabR	5'-gcgtacgtaCTATCTCCTTTCCTTTGCCTTGTC-3'
AtERF13entrF	5'-CACCATGAGCTCATCTGATTCCG-3'
AtERF13entrR	5'-gcgactagtTTATATCCGATTATCAGAATAAGAACATTC-3'
eGFPentrF	5'-CACCATGGTGAGCAAGGGCGAGGAGC-3'
eGFPR	5'-gcgctcgagTTACTTGTACAGCTCGTCCATGCCGAG-3'
TGMVcleFBgl	5'-gcgggtacc***TTAGATCT***TTAGACGACAATGCGGCGT-3'
TGMVSacIR	5'-gcggagctcTCTAGAAGTACTAGTACTGC-3'
TGMVcleRBgl	5'-gcg***AGATCTTT***GCATATGTGAAGGGCCAAT-3'
TGMVKpnF	5'-gcgggtaccGAATTCCAGGTCGACGGTCGAAG-3'
CabcleXhoF	5'-gcg***CTCGAGA***GCATTAAATGAAATCTAACCAATC-3'
CabSacIR	5'-gcggagctcTCCTGGCGTCCTCCCCTGGAG-3'
CabcleXhoR	5'-gcg***CTCGAGT***ACGCAAGACACCAAAAAAAT-3'
CabdIIIF	5'-gcgaagcttGAATTCTCTGCCTGAGCTAC-3'
AtTCP24BamNcoF	5'-gcgggatccATGGAGGTTGACGAAGACATTG-3'
AtTCP24XhoR	5'-gcgctcgagCTATCTCCTTTCCTTTGCC-3'
AL2FBgl	5'-gcgagatctATGCGAAATTCGTCTTCCTC-3'
AL2INTEIN3'	5'-gcggaattcCTATTTAAATAAGTTCTC-3'
GSTPacIF	5'-gcgttaattaaATGTCCCCTATACTAGGTTA-3'
GSTSnaNotR	5'-gcgGCGGCCGCCacTACGTAccacgcggaaccagatccg-3'
AtTCP24NotIR	5'-gcggcggccgcCTATCTCCTTTCCTTTGCCTTG-3'
AtERF13NotIR	5'-gcggcggccgcTTATATCCGATTATCAGAATAAG-3'
3xFLAGPacF	5'-gcgttaattaaATGGATTACAAGGACCAC-3'
TGMVAL2NotIR	5'-gcggcggccgcCTATTTAAATAAGTTCTC-3'
TGMVAL1NcoF	5'-GCGCCATGGGGCTTACTACCAATTG-3'
TGMVCREcoR	5'-gcggaattcTTTGAATTAAAGATCCACG-3'
TGMVIRrfitc	5'-[FI]TTTGAATTAAAGATCCACG-3'
CbLCVCCRKpnF	5'-gcgggtaccTTTTGTAAATAATATTTAGGAC-3'
CbLCVCCREcoR	5'-gcggaattcAAATTGAAATCACATGTC-3'
CaLCuVIRAcpFITC	5'-[FI]AAATTGAAATCACATGTC-3'
TGMVRP7v	5'-GCCATTCTCTGCTTGAGGCACAGTGATAGG-3'
TGMV RP5c	5'-GAATTGACCTGAACTGTGGCTGTTCCATAT-3'
CLCVRep5	5'-GGATTTGGAACCTCCCAG-3'
CLCVRep3	5'-GTACGCCGAAGACGCATTG-3'
M13F	5'-GTAAAACGACGGCCAG-3'
M13R	5'-CAGGAAACAGCTATGAC-3'
TGMVCPReg5	5′-gcggaattcCCGGATGGCCGCGCGATCGTC-3′
TGMVCPReg3	5′-gcggaattcCTTAGATTTCAGGGCC-3′
CRA2F2	5'-gcgctcgagccatggGTAATTAAGAGGCTTACTACC-3'
GUSSeq	5'-CCCACCAACGCTGATCAATTCCAC-3'
GUSR	5'-gcgctcgagTCATTGTTTGCCTCCCTGC-3'

TGMVqPCRNbF	5'- AGAATCACACGAACAGTGTC -3'
TGMVqPCRNbR	5'- TGAACACTTGTCCGAAATCC -3'
CaLCuVqPCRNbF	5'- CAAGAAGCAGGGAAATACGAG -3'
CaLCuVqPCRNbR	5'- GTCGCATACACAGGATTAGAG -3'
CaBqPCRAtF	5'- CGGGCTGCTAAACGAAACGATTTAGGGTTTC-3'
CaBqPCRAtR	5'- ATAGGTTTTAGAGAGAGCTCTCCTGGCGTC-3'
CaLCuV Amp AF	5'- TCCTTGATGTTCGACTTTCC-3'
CaLCuV Amp AR	5'- AACCCACATACATCAGAGTCGC-3'
CaLCuV Amp BF	5'- CGGGCTGCTAAACGAAACGATTTAGGGTTTC-3'
CaLCuV Amp BR	5'- ATAGGTTTTAGAGAGAGCTCTCCTGGCGTC-3'
CaLCuV Amp CF	5'- CCATCCGCAATAATATTACCG-3'
CaLCuV Amp CR	5'- AAATCACATGTCTTTATATAGGCCC-3'
CaLCuV Amp DF	5'- GTGATTTCAATTTATGCTTTACTTCG-3'
CaLCuV Amp DR	5' AGCGGCCTTATCAAATTTATG-3'
CaLCuV Amp EF	5'- ACCTCGTGCAGGTATGATCC-3'
CaLCuV Amp ER	5'- ACGTCCTATAAATCCTGGGC-3'
TGMV Amp 1F	5'- CCCATCTTCATGAAGCTCTCTGC-3'
TGMV Amp 1R	5'- CTTACATATCCTCAGTGCTCCTTGTCC-3'
TGMV Amp 2F	5'- TTGGCATTTATTTGAAACCG-3'
TGMV Amp 2R	5'- GGAACTTCTAATATATAAGAGCTACCTTACTACC-3'
TGMV Amp 3F	5'- CGTGGATCTTTAATTCAAAATGCC-3'
TGMV Amp 3R	5'- ACGCTTAGGCAAACTTCCTCG-3'
TGMV Amp 4F	5'- CCCAGGATATATCGATCACTAAGAGG-3'
TGMV Amp 4R	5'- ACATCAGATATACACATGACCTTCCC-3'
18S F	5'- AAACGGCTACCACATCCAAG-3'
18S R	5'- ACTCGAAAGAGCCCGGTATT-3'
PP2A F	5'- TATCGGATGACGATTCTTCGTGCAG-3'
PP2A R	5'- GCTTGGTCGACTATCGGAATGAGAG-3'
TCP2FP	5'- TCAGGTGGTGGCTTCAGTGG-3'
TCP2RP	5'- ACCTCCTCCACTTTCTGATGTTGG-3'
TCP3FP	5'- CTCCGTCGTCGTTGCATCC-3'
TCP3RP	5'- TGATGATGATGGTGAGGATCAAACC-3'
TCP4FP	5'- GTCGTTTCAGCCAGTTCTTGG-3'
TCP4RP	5'- TGATGATGGTGAGGATCAAACC-3'
TCP10FP	5'- GCCAACAACAACAACAAAGG-3'
TCP10RP	5'- TGATGAAGCAAACATCGAAGAAGC-3'
TCP24FP	5'- CACCTCCACCTCTTGACCAC-3'
TCP24RP	5'- TCATCAGAGCTCCGGTTTCC-3'
CaLCuV B-IR RightF	5'- GGCTAACGCTTATCTCGGTGTTA- 3'
CaLCuV B-IR RightR	5'- TAACTCCGTAGAGGAAATATAATTGGG-3'

The primer sequence is given in the 5′ to 3′ direction and viral sequences that have been mutated are shown in bold and italicized. Lower case letters indicate additional sequences at the 5'-end of the primer with any restriction site added for cloning purposes underlined.
